# BAF155 promotes cardiac hypertrophy and fibrosis through inhibition of WWP2-mediated PARP1 ubiquitination

**DOI:** 10.1038/s41421-023-00555-x

**Published:** 2023-05-08

**Authors:** Naijin Zhang, Ying Zhang, Yong Chen, Hao Qian, Boquan Wu, Saien Lu, Shilong You, Wancheng Xu, Yuanming Zou, Xinyue Huang, Wenbin Wang, Jingwei Liu, Da Li, Liu Cao, Yingxian Sun

**Affiliations:** 1 Department of Cardiology, First Hospital of China Medical University, Shenyang, Liaoning China; 2 Key Laboratory of Reproductive and Genetic Medicine, National Health Commission, China Medical University, Shenyang, Liaoning China; 3 State Key Laboratory of Molecular Biology, Shanghai Institute of Biochemistry and Cell Biology, Center for Excellence in Molecular Cell Science, Chinese Academy of Sciences, Shanghai, China; 4 Institute of School of Basic Medicine, China Medical University, Shenyang, Liaoning China; 5 Center of Reproductive Medicine, Shengjing Hospital of China Medical University, Shenyang, Liaoning China; 6 Key Laboratory of Environmental Stress and Chronic Disease Control and Prevention, Ministry of Education, China Medical University, Shenyang, Liaoning China

**Keywords:** Ubiquitylation, Molecular biology

Dear Editor,

BAF155 is a subunit of the SWI/SNF chromatin remodeling complexes, which increase DNA accessibility by remodeling nucleosomes during gene transcription^1^. BAF155 plays an important role in development and disease. For instance, the BAF155-containing BAF complex is required to maintain self-renewal and pluripotency of embryonic stem cells through regulation of Oct4/Sox2-dependent transcription^2^. BAF155 has also been shown to promote breast cancer progression and metastasis by regulating the expression of c-Myc pathway genes^3,4^. However, the roles and mechanisms of BAF155 in cardiovascular disease remain unknown. Here, we found that BAF155 expression was notably upregulated in cardiac tissues of patients and mice with heart failure and in angiotensin II (Ang II)-treated cardiomyocytes (Fig. 1a; Supplementary Fig. S1a, b).

**Fig. 1:**
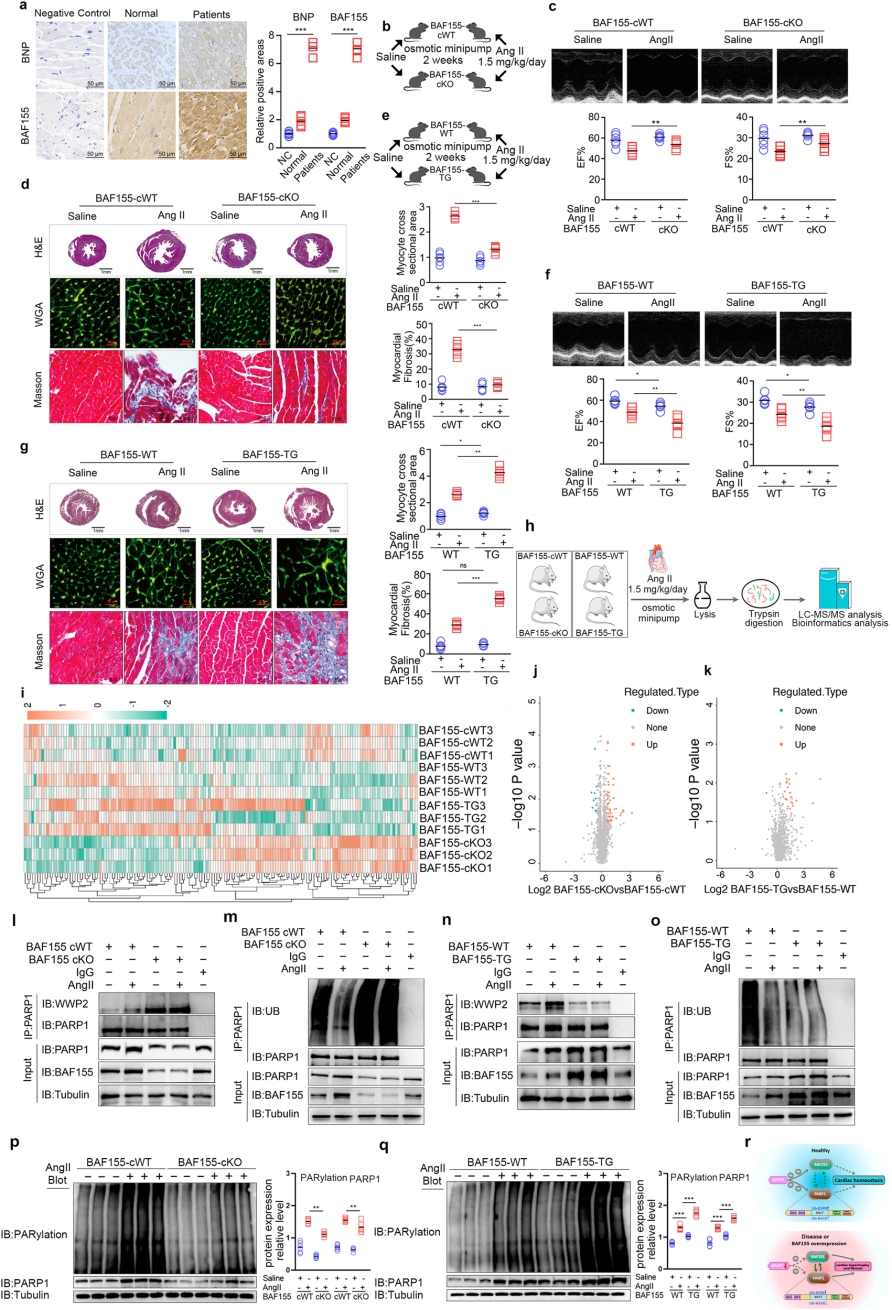
BAF155 restrains WWP2-mediated PARP1 ubiquitination to promote cardiac hypertrophy and fibrosis. **a** Immunohistochemical staining of BAF155 and BNP proteins in heart tissues from healthy donors and patients with heart failure (*n* = 5). **b** Schematic of Ang II-induced mouse model of cardiac hypertrophy and fibrosis. *BAF155*-cWT and *BAF155*-cKO mice were administered with saline or Ang II (1.5 mg/kg/day) through a subcutaneously implanted osmotic minipump (0.5 µL/h) for 2 weeks. **c** EF% and FS% of *BAF155*-cWT and *BAF155*-cKO mice (*n* = 6). **d** H&E staining, TRITC-labeled WGA staining, and Masson’s trichrome staining of *BAF155*-cWT and *BAF155*-cKO hearts (*n* = 6). **e** Schematic of Ang II-induced mouse model of cardiac hypertrophy and fibrosis. *BAF155*-WT and *BAF155*-TG mice were administered with saline or Ang II (1.5 mg/kg/day) through a subcutaneously implanted osmotic minipump (0.5 µL/h) for 2 weeks. **f** EF% and FS% of *BAF155*-WT and *BAF155*-TG mice (*n* = 6). **g** H&E staining, TRITC-labeled WGA staining, and Masson’s trichrome staining of *BAF155*-WT and *BAF155*-TG hearts (*n* = 6). **h** Schematic showing workflow for quantitative proteome analysis. **i** Heatmaps of differentially expressed proteins among *BAF155*-cWT, *BAF155*-cKO, *BAF155*-WT, and *BAF155*-TG hearts (*n* = 3). **j**, **k** Volcano plots showing foldchanges of all detected proteins between *BAF155*-cWT and *BAF155*-cKO hearts (**j**), and *BAF155*-WT and *BAF155*-TG hearts (**k**). **l** Co-IP assay showing the interaction between WWP2 and PARP1 in *BAF155*-cWT and *BAF155*-cKO hearts. **m** Ubiquitination levels of PARP1 in *BAF155*-cWT and *BAF155*-cKO hearts. **n** Co-IP assay showing the interaction between WWP2 and PARP1 in *BAF155*-WT and *BAF155*-TG hearts. **o** Ubiquitination levels of PARP1 in *BAF155*-WT and *BAF155*-TG hearts. **p**, **q** Immunoblotting showing expression of PARP1 and levels of total PARylation in *BAF155*-cWT and *BAF155*-cKO hearts (**p**), and *BAF155*-WT and *BAF155*-TG hearts (**q**) (*n* = 6). **r** Working model showing the role of BAF155 in regulating cardiac homeostasis. Data represent means ± SD. *P < 0.05, **P < 0.01, ***P < 0.001. Two-way ANOVA with Bonferroni multiple comparisons test (**a**, **c**, **d**, **f**, **g**, **p**, **q**).

To explore the effect of BAF155 on heart disease, we generated conditional myocardium-specific *Myh6*^*Cre+*^;*BAF155*^*Fl/Fl*^ (hereafter, *BAF155*-cKO) mice, and *Myh6*^*Cre–*^*;BAF155*^*Fl/Fl*^ (hereafter, *BAF155*-cWT) mice were used as controls. We established a mouse model of cardiac hypertrophy and fibrosis by Ang II (1.5 mg/kg/day) infusion for 14 days (Fig. 1b; Supplementary Fig. S1c, d)^5,6^. Notably, compared with *BAF155*-cWT mice, *BAF155*-cKO mice displayed significantly alleviated Ang II-induced cardiac dysfunction, as reflected by increased ejection fraction (EF%) and fractional shortening (FS%) (Fig. 1c). Cardiac hypertrophy and fibrosis were also significantly mitigated in *BAF155*-cKO mice as revealed by hematoxylin and eosin (H&E), wheat germ agglutinin (WGA), scanning-source optical coherence tomography and Masson staining assays (Fig. 1d; Supplementary Figs. S1e, f, S2a). We found that compared to *BAF155*-cWT mice, expression levels of cardiac hypertrophy and heart failure markers (ANP and BNP), fibrosis markers (α-SMA and Col-1), cardiomyocyte death markers (cleaved-caspase-3, cleaved-caspase-9), and DNA damage response markers (p-ATM and p-ATR) were significantly decreased in *BAF155*-cKO mice (Supplementary Fig. S1g–j). Since *Myh6-Cre* is active in both cardiomyocytes and smooth muscle cells^7^, arteries were further collected for histology analyses. We found that knockout of *BAF155* mitigated Ang II-induced hypertension, vascular thickening, and fibrosis (Supplementary Fig. S2b–e). These results suggested that *Myh6-Cre*-induced knockout of *BAF155* in mice alleviated Ang II-induced cardiac hypertrophy and fibrosis, which improved heart function by modulating myocardial cells and reduced hypertension by modulating vascular smooth muscle cells.

To further examine whether BAF155 overexpression in cardiomyocytes can aggravate heart disease, we generated transgenic mice overexpressing BAF155 driven by the CAG promoter (hereafter, *BAF155*-TG) (Fig. 1e; Supplementary Fig. S3a). Notably, compared with *BAF155*-WT mice, *BAF155*-TG mice displayed a slight reduction in EF% and FS% under physiological conditions, and Ang II treatment further aggravated the impairment of EF% and FS%, as demonstrated by echocardiography imaging (Fig. 1f). Likewise, under physiological conditions, *BAF155*-TG mice displayed mild cardiac hypertrophy, which was significantly aggravated after Ang II treatment (Fig. 1g; Supplementary Figs. S3b, c, S4a). At the molecular level, under physiological conditions, *BAF155*-TG mice exhibited slightly increased expressions of α-SMA, cleaved-caspase-3, cleaved-caspase-9, p-ATM, and p-ATR compared with *BAF155*-WT mice. Moreover, compared with *BAF155*-WT mice, the expression levels of ANP and BNP were significantly increased in Ang II-treated *BAF155*-TG mice (Supplementary Fig. S3d–g). We also found that *BAF155*-TG mice displayed aggravated Ang II-induced hypertension, vascular thickening, and fibrosis (Supplementary Fig. S4b–e). These results confirmed that Ang II-induced cardiac hypertrophy and fibrosis were aggravated in *BAF155*-TG mice.

To identify the potential target of BAF155, cardiac tissues from Ang II-treated *BAF155*-cWT, *BAF155*-cKO, *BAF155*-WT, and *BAF155*-TG mice were collected for quantitative proteomic analysis (Fig. 1h). A total of 3982 proteins were identified (Supplementary Figs. S5, S6a–g), and differentially expressed proteins were shown in Fig. 1i–k. We further identified potential protein interactors of BAF155 using mass spectroscopy. Interestingly, we found that Poly(ADP-ribose) polymerase 1 (PARP1), a known player in cardiac hypertrophy and fibrosis, was a potential interacting protein of BAF155 (Supplementary Fig. S7 and Table S1). Co-IP assays confirmed the interaction between BAF155 and PARP1, which was increased with Ang II treatment (Supplementary Fig. S6h–j). Co-IP assays using a series of truncation variants suggested that BAF155 mainly interacted with amino acids 1–203 (Zinc finger domains 1/2) and 476–779 (PARP-A-helical domain) of PARP1 (Supplementary Fig. S6k).

We next explored the mechanism by which PARP1 is regulated by BAF155. As shown in Supplementary Fig. S8a, BAF155 overexpression resulted in increased expression of PARP1, whereas BAF155 silencing remarkably downregulated the expression of PARP1 (Supplementary Fig. S8b). In addition, treatment with cycloheximide (CHX), a protein translation inhibitor, resulted in decreased expression of PARP1 in a dose-dependent manner in control cells, whereas BAF155 overexpression could maintain PARP1 abundance in the presence of CHX (Supplementary Fig. S8c). Furthermore, treatment with MG132, a proteasome inhibitor, dose-dependently increased the expression of PARP1 in control cells; however, BAF155 overexpression maintained a high level of PARP1 expression (Supplementary Fig. S8d). Altogether, these findings indicated that BAF155 inhibited PARP1 degradation by blocking the proteasome pathway. Consistently, the ubiquitination level of PARP1 was decreased in BAF155-overexpressing cells (Supplementary Fig. S8e), whereas the ubiquitination level of PARP1 was increased by BAF155 knockdown (Supplementary Fig. S8f).

Our previous study found that K249 and K418 were key sites of PARP1 ubiquitination^8^. To further explore whether PARP1 ubiquitination was inhibited by BAF155, we overexpressed *PARP1*-WT, *PARP1*-K249R, or *PARP1*-K418R in control cells or BAF155 knockdown cells. As shown in Supplementary Fig. S8g, compared with the *PARP1*-WT, ubiquitination levels of the *PARP1*-K249R, *PARP1*-K418R were decreased in control cells, and BAF155 knockdown remarkably increased ubiquitination levels of *PARP1*-WT, but not *PARP1*-K249R or *PARP1*-K418R. Collectively, these results demonstrated that BAF155 might inhibit PARP1 ubiquitination at K249 and K418 sites.

Our previous work revealed that WWP2 is a specific E3 ubiquitination ligase of PARP1 and mediates the ubiquitination of PARP1 at K249 and K418 sites^8^. WWP2 is also an E3 ubiquitination ligase of BAF155^9^. Treatment with MG132 enhanced the interaction between BAF155 and WWP2, as well as that between PARP1 and WWP2 (Supplementary Fig. S8h). Furthermore, upon BAF155 knockdown, binding between PARP1 and WWP2 was increased (Supplementary Fig. S8i), whereas BAF155 overexpression decreased interaction between PARP1 and WWP2 (Supplementary Fig. S8j). When WWP2 was overexpressed, the ubiquitination level of PARP1 was decreased by overexpression of BAF155, compared with the control group (Supplementary Fig. S8k).

We further examined the regulation of PARP1 by BAF155 in the established mouse model of Ang II-induced cardiac hypertrophy and fibrosis. Our results showed that under Ang II treatment, the interaction between PARP1 and WWP2 was enhanced in cardiac tissues from *BAF155*-cKO mice compared with *BAF155*-cWT mice (Fig. 1l). In addition, under Ang II treatment, PARP1 ubiquitination was also increased in *BAF155*-cKO mice (Fig. 1m). In contrast, compared with *BAF155*-WT mice, the interaction between PARP1 and WWP2 was downregulated in *BAF155*-TG mice with Ang II treatment (Fig. 1n). The ubiquitination level of PARP1 was also decreased in cardiac tissues of *BAF155*-TG mice with Ang II treatment (Fig. 1o).

Interestingly, WWP2 may target the BAF155–PARP1 complex to regulate its ubiquitination and degradation, thereby protecting the heart against hypertrophy and fibrosis. To further understand the role of WWP2 in Ang II-induced cardiac hypertrophy and fibrosis, we generated *WWP2*-cKO and *WWP2*-cWT mice. Proteomic analysis for global ubiquitination was performed (Supplementary Figs. S9, S10a–h). Co-IP assays showed that the interaction between BAF155 and PARP1 was increased in *WWP2*-cKO mice with Ang II treatment, compared with *WWP2*-cWT mice (Supplementary Fig. S10i). In addition, PARP1 ubiquitination was downregulated in *WWP2*-cKO mice with Ang II treatment (Supplementary Fig. S10j). BAF155 ubiquitination was also decreased in *WWP2*-cKO mice with Ang II treatment (Supplementary Fig. S10k). These results suggested BAF155–PARP1 as a key physiological substrate of WWP2 in vivo.

PARP1 is an abundant nuclear protein involved in various DNA repair pathways. Previous studies have shown that PARP1 promotes cardiac hypertrophy by PARylation of downstream targets, including BRD4, HMGB1, CEBPβ, and FOXO3a^10–12^. We next examined the role of BAF155 in the regulation of PARP1 expression and total protein PARylation levels. We found that *BAF155*-cKO mice displayed decreased Ang II-induced expression of PARP1, total PARylation modification, and PARylation levels of BRD4, HMGB1, CEBPβ, and FOXO3a, compared with *BAF155*-cWT mice (Fig. 1p; Supplementary Fig. S11a–d). In contrast, compared with those in *BAF155*-WT mice, expression of PARP1, total PARylation modification, and PARylation levels of BRD4, HMGB1, CEBPβ, and FOXO3a were mildly increased in *BAF155*-TG mice under physiological conditions, which were significantly increased following Ang II stimulation (Fig. 1q; Supplementary Fig. S11e–h).

To determine whether PARP1 inhibition can restrain the detrimental effects of BAF155 overexpression on cardiac function, a PARP1 inhibitor (25 mg/kg/day for 14 days) was administered to Ang II-treated *BAF155*-TG mice (Supplementary Fig. S12a). Remarkably, PARP1 inhibition significantly improved cardiac EF% and FS% and alleviated cardiac hypertrophy and fibrosis (Supplementary Fig. S12b–d). Consistently, PARP1 inhibitor treatment downregulated expression levels of PARP1, cleaved-caspase-3, cleaved-caspase-9, p-ATM, p-ATR, and total PARylation levels in *BAF155*-TG mice with Ang II treatment (Supplementary Fig. S12e–g).

Collectively, our study shows that BAF155 promotes pathological cardiac hypertrophy and fibrosis by inhibiting WWP2-mediated PARP1 ubiquitination and degradation (Fig. 1r). Therefore, our findings suggest BAF155 as a potential target in the treatment of myocardial hypertrophy and fibrosis disease.

## Supplementary information

Supplementary information
