# Supplementary material for: BAF155 promotes cardiac hypertrophy and fibrosis through inhibition of WWP2-mediated PARP1 ubiquitination
Source: Cell Discov. 2023 May 8;9:46. doi: 10.1038/s41421-023-00555-x (PMC10167234; doi:10.1038/s41421-023-00555-x)
Supplement: Supplementary file 1 — Supplementary information [file 41421_2023_555_MOESM1_ESM.pdf]

Supplementary information for

BAF155 promotes cardiac hypertrophy and fibrosis through inhibition of WWP2-mediated PARP1 ubiquitination

Naijin Zhang[1,2], Ying Zhang[1], Yong Chen[3], Hao Qian[1], Boquan Wu[1], Saien Lu[1], Shilong You[1], Wancheng Xu[3], Yuanming Zou[1], Xinyue Huang[1], Wenbin Wang[1], Jingwei Liu[4], Da Li[2,5], Liu Cao[4], Yingxian Sun[1,6]*

*** Corresponding author:** Yingxian Sun (yxsun@cmu.edu.cn)

[#] These authors contributed equally: Naijin Zhang, Ying Zhang

[1]Department of Cardiology, First Hospital of China Medical University, Shenyang, Liaoning, China.

[2]Key Laboratory of Reproductive and Genetic Medicine, National Health Commission, China Medical University, Shenyang, Liaoning, China.

[3]State Key Laboratory of Molecular Biology, Shanghai Institute of Biochemistry and Cell Biology, Center for Excellence in Molecular Cell Science, Chinese Academy of Sciences, Shanghai, China.

[4]Institute of School of Basic Medicine, China Medical University, Shenyang, Liaoning, China.

[5]Center of Reproductive Medicine, Shengjing Hospital of China Medical University, Shenyang, Liaoning, China.

[6]Key Laboratory of Environmental Stress and Chronic Disease Control and Prevention, Ministry of Education, China Medical University, Shenyang, Liaoning, China.

This file includes:

Materials and Methods

Supplementary References

Supplementary Figures S1 to S12

Supplementary Tables S1 to S3

Materials and Methods

1. Ethics approval

The study included five male patients and five age- and gender-matched controls. Failing heart samples were obtained from patients with end-stage heart failure (average ejection fraction was 20 ± 5%) at the time of cardiac transplantation (Northern Theater Command General Hospital). Nonfailing hearts were obtained from donors who had normal cardiac contractile function by echocardiography or anatomical analysis of remains and had died from accidents (Department of Forensic Medicine, China Medical University; Center of Organ Transplantation, The First Hospital of China Medical University). The procurement of the heart tissues conforms to the principles outlined in the Declaration of Helsinki and was approved by the Institutional Ethics Committee of The First Hospital of China Medical University (protocol number AF-SOP-07-1.1-01; [2022]506).

2. Immunohistochemistry for human hearts tissues from normal and heart failure patients

Heart tissues were fixed in neutral buffered formalin solution, embedded in paraffin, and then cut into 5-um serial sections. Sections were dewaxed in xylene, rehydrated in a series of descending percentages of ethanol and boiled in Tris-EDTA solution for antigen retrieval. UltraSensitiveTM SP IHC Kit (MXB, China) was used for immunostaining. After blocking, the slides were incubated with primary antibody for BNP (Abcam, USA) and BAF155 at 37°C for 1 h. After washing, the sections were immersed with biotin-conjugated IgG secondary antibody in the IHC kit for 20 min, then with DAB (MXB, China) for 2 min, and then stained with hematoxylin for 2 min. Immunohistochemical staining was semiquantitated by determining the percentage of cardiomyocytes stained using 10% increments (0%, 10%, 20%, 30%, …, 100%) as in a previous study [1]. The mean values from two evaluators were used for statistical analysis.

3. BAF155 and WWP2 knockout, and transgenic mice

Conditional cardiomyocyte specific knockout (KO) mice, including *Myh6*$^{Cre+}$; *BAF155* $^{Fl/Fl}$ (*BAF155*-cKO) and *Myh6*$^{Cre-}$; *BAF155* $^{Fl/Fl}$ (*BAF155*-cWT), *BAF155*-WT and *BAF155* transgenic (*BAF155*-TG) mice (CAG promoter) were obtained from Shanghai Model Organisms Science and Technology Development, as well as conditional cardiomyocyte specific knockout mice, including *Myh6*$^{Cre+}$, *WWP2*$^{Fl/Fl}$ (*WWP2*-cKO) and *Myh6*$^{Cre-}$,

WWP2$^{Fl/Fl}$ (*WWP2*-cWT) animals. In this study, 8 to 10-week-old specific pathogen-free (SPF) male mice were included. In angiotensin II (Ang II) (1.5 mg/kg/day) and NaCl infusion mouse models, including *BAF155*-cKO, *BAF155*-cWT, *BAF155*-WT, *BAF155*-TG, *WWP2*-cKO and *WWP2*-cWT mice (6 per group for totally n=72), and NaCl and PARP1 inhibitor (Veliparib, ABT-888, 25 mg/kg/d) infusion mouse models, including *BAF155*-TG, *BAF155*-TG with Ang II (6 per group for totally n=36), were randomly assigned to groups and submitted to anesthesia (isoflurane at 2% in oxygen; 1,500 ml/min). This was followed by incision in the middle scapular region and subcutaneous implantation of an osmotic minipump (Alzet) as directed by the manufacturer [2-3]. During the following 14 days, mouse euthanasia was performed by cervical dislocation. All animal experiments were approved by the Animal Subjects Committee of China Medical University and performed in accordance with the U.S. National Institutes of Health (NIH) Guide for the Care and Use of Laboratory Animals (publication no. 85-23, 1996).

4. Proteomics and ubiquitination proteomics

4.1. Protein extraction

Specimens were pulverized in liquid nitrogen and placed in 5-mL tubes, Supplementaryed four volumes of lysis buffer (8 M urea, 1% Protease Inhibitor Cocktail) and sonicated three times on ice with a Scientz ultrasonic homogenizer. This was followed by centrifugation-clearing at 12,000 g and 4°C for 10 min. Protein concentration of the resulting supernatant was measured using BCA kit.

4.2. Trypsin digestion

Equal quantity of total protein in each sample was enzymatically digested, and the volume was adjusted to be consistent across the sample set. TCA was added dropwise to a final concentration of 20%, followed by vortex mixing and precipitation at 4°C for 2h. Centrifugation was performed at 4500 g for 5 min, and the resulting pellet was washes with pre-cooled acetone twice. Followed by drying of pellet, pellet was resuspended by 200mM TEAB facilitated by sonication, trypsin was added to each sample at 1:50 (protease: protein, w/w) for overnight digestion. Dithiothreitol (DTT) was added at 5 mM followed by incubation at 56°C for 30 min. Then, iodoacetamide (IAA) at 11 mM was added followed by incubation for 15 min at room temperature at darkness.

4.3. Enrichment of post-translational modified peptides

Peptides were dissolved in IP buffer (100 mM NaCl, 1 mM EDTA, 50 mM Tris-HCl, 0.5% NP-40, pH 8.0), mixed with pre-washed anti-ubiquitin remnant antibody resins (PTM-1104; Hangzhou Jingjie PTM-Bio), and incubated with gentle shaking overnight at 4°C. Antibody resin was washed with IP buffer and deionized water, respectively. Finally, enriched peptides were eluted with 0.1% trifluoroacetic acid three times and cleaned by C18 ZipTips.

4.4. LC-MS/MS analysis

Tryptic peptides were dissolved in liquid chromatographic mobile phase A and separated on a NanoElute ultra-high performance liquid system. Mobile phases A and B were 0.1% formic acid and 2% acetonitrile in water and 0.1% formic acid in acetonitrile, respectively. Peptides were eluted using the gradient with a constant flow rate at 450 nL/min. The elution gradient was set as: 0-72 min, 7%-24% B; 72-84 min, 24%-32% B; 84-87 min, 32%-80% B; 87-90 min, 80% B. Peptides were separated on a capillary column (inner ID, particle size) before injecting into a capillary ion source for ionization and TIMS-TOF Pro mass spectrometer (ion source voltage, 1.6 kV; scanning range, 100-1700 Da). The parallel accumulation serial fragmentation (PASEF) mode was enabled for data acquisition. Precursors with charge states 0 to 5 were selected for fragmentation, and 10 PASEF MS/MS scans were acquired per cycle. The dynamic exclusion time of MS/MS scanning was 30 seconds to prevent multiple scanning of the same parent ions.

4.5. Database search

Raw mass spectrometry data was searched against a Swissprot protein sequence database (Mus_musculus_10090_SP_20201214.fasta) by Maxquant (v1.6.15.0) with reverse decoy entries and common contamination proteins. A maximum of 2 missing cleavages was allowed for Trypsin/P digestion, and at least 7 amino acids were required for each peptide. Mass error tolerance for precursor ion was 10 ppm and product ion was 20 ppm, respectively. Cysteine alkylation [carbamidomethyl (C)] was considered a fixed modification. Variable modifications were methionine oxidation and n-terminal acetylation. Lysine ubiquitination and di-glycine on lysine were also set as variable modification for corresponding modification enrichment analysis. The FDRs for protein and PSM identifications were both 1%.

4.6. Proteomics quantification (relative value)

The raw LC-MS datasets were first searched against database and converted into matrices containing LFQ intensity (the raw intensity after correcting the sample/batch effect) of proteins. The LFQ intensity (I) was transformed to the relative quantitative value (R) after centralization. The formula is listed as follow where i represents sample and j represents protein:

$$R_{ij} = I_{ij} / \mathrm{Mean}(I_j)$$

4.7. Differential screening of proteomics (fold change)

Firstly, the samples to be compared were selected in pairwise groups, and the fold change (FC) was then calculated by the ratio of the mean intensity for each protein in two sample groups. For example, to calculate the fold change between sample A and sample B, the formula is shown as following: R denotes the relative quantitative value of the protein, i denotes the sample and k denotes the protein.

$$FC_{A/B,k} = \mathrm{Mean}(R_{ik}, i \in A) / \mathrm{Mean}(R_{ik}, i \in B)$$

To calculate the statistical significance of difference between groups, the student's T test was performed on the relative quantitative value of each protein from the two sample groups. The corresponding P value was calculated as the significance index. The relative quantitative value of proteins was applied with log2 transformation typically. The formula is shown as following:

$$P_{ik} = \mathrm{T.test}(\mathrm{Log2}(R_{ik}, i \in A), \mathrm{Log2}(R_{ik}, i \in B))$$

The protein with P value < 0.05, the fold change > 1.5 was regarded as significantly up-regulated protein, while the protein with *P* value < 0.05, the fold change $< 1/1.5$ was regarded as significantly down-regulated protein. (*P* value < 0.05, the fold change > 1.5).

4.8. Gene Ontology (GO) analysis

UniProt-GOA database (www.http://www.ebi.ac.uk/GOA/) was utilized for GO annotation. First, obtained protein identifications were mapped to GO IDs based on their uniport IDs. For proteins with no annotation in UniProt-GOA, InterProScan was utilized for annotating GO functions based on the protein sequence alignment. Proteins were assigned to biological process, cellular component and molecular function as GO terms. In various categories, a two-tailed Fisher's test was utilized for assessing the enrichment of differentially expressed proteins

versus all detected proteins; corrected *P*<0.05 indicated statistical significance.

4.9. Subcellular localization

Wolfpsort, latest version of PSORT/PSORT II, was utilized for predicting the subcellular localization of eukaryotic proteins.

4.10. Proteomic quality control of *BAF155*-cWT, *BAF155*-cKO and *BAF155*-WT, *BAF155*-TG mice

For the biological repeatability of omics results, we determined that the quantitative data of biological or technical duplicates were statistically consistent. To this end, Principal Component Analysis (PCA) (Supplementary Figure S5a), Pearson's correlation analysis (Supplementary Fig. S5b) and Relative Standard Deviation (RSD) determination (Supplementary Fig. S5c) were carried out. Supplementary Figure S5d demonstrates the total spectra and the numbers of peptides and quantifiable proteins detected by BAF155 omics mass spectrometry. The protein molecular weight distribution diagram (Supplementary Fig. S5e) shows the molecular weights of proteins obtained by BAF155 proteomics. The peptide number distribution map (Supplementary Fig. S5f) showed that most proteins corresponded to more than two peptides, suggesting the accuracy and credibility of BAF155 omics results.

4.11. Proteomic quality control of *WWP2*-cWT and *WWP2*-cKO mice

PCA (Supplementary Fig. S11a), Pearson's correlation analysis (Supplementary Fig. S11b) and RSD determination (Supplementary Fig. S11c) were carried out for evaluating protein quantitation repeatability in *WWP2*-cWT and *WWP2*-cKO mouse heart tissues. Supplementary Fig. S11d demonstrates the total spectra, and the numbers of peptides and quantifiable proteins detected by WWP2 omics mass spectrometry. The protein molecular weight distribution diagram (Supplementary Fig. S11e) shows the molecular weights of proteins obtained by WWP2 proteomics. The peptide number distribution map (Supplementary Fig. S11f) showed that most proteins corresponded to more than two peptides, indicating the accuracy and credibility of WWP2 omics results. The proteins' molecular weights had a positive correlation with coverage (Supplementary Fig. S11g). For achieving a comparable coverage, a greater number of peptides must be identified for larger proteins. The coverage of most proteins was below 30% (Supplementary Fig. S11h).

5. Histopathological assessment

Myocardial tissue and aorta abdominal samples underwent formalin (4%) fixation for 4 h, paraffin embedding and sectioning at 5 μm. After xylene dewaxing, rehydration was carried out with graded ethanol followed by subsequent hematoxylin and eosin (H&E) and Masson's trichrome (G1340; Solarbio, China) staining.

Frozen myocardial tissue sections were examined by immunofluorescence. Cross-sectional areas of cardiomyocytes were assessed in images obtained after staining with 5 μM wheat germ agglutinin (WGA) (Thermo, USA).

6. Echocardiography and left ventricular function (LVEF) assessment

Heart function in the *Myh6*$^{Cre+}$*, BAF155*$^{Fl/Fl}$; *Myh6*$^{Cre-}$, *BAF155*$^{Fl/Fl}$, *BAF155*-WT and *BAF155*-TG groups were assessed on a Visual Sonics Vevo 2100 real-time, high-resolution *in vivo* micro-imaging system (Visualsonic, Canada; VINNO6 Lab, China). Totally 48 mice were anesthetized with 1.5% isoflurane, followed by cardiac function analysis by a 40 MHz transducer with continuous oxygen supply. LVEF was examined by two-dimensional M-mode recording. Heart function determination was based on interventricular septal dimension (IVSd), left ventricular posterior wall dimension (PWTd), LV internal dimension at systole (LVDs), LV internal dimension at diastole (LVDd) and LV mass measurements. Additionally, left ventricular ejection fraction (EF%) and fractional shortening (FS%) were determined.

7. Cells and treatment

HEK293T and H9c2 cells (ATCC) were cultured in high-glucose Dulbecco's modified Eagle's medium containing 10% FBS (HyClone), at 37°C in a humidified 5% CO_2 incubator.

8. Plasmid construction, antibodies and reagents

Supplementary Table S2 listed various plasmids and small hairpin RNA (shRNAs). Lipofectamine 3000 (Invitrogen, USA) was utilized for transfection. Lentiviruses were used for shRNA transduction. Supplementary Table S3 listed all antibodies used in the study. MG132 (A2585), a proteasome inhibitor, and cycloheximide (CHX, A8244) were obtained from Apexbio (USA) and dissolved with DMSO. Ang II (A9525; Sigma, USA) in DMSO was utilized at 10 μM.

9. PARP1 and BAF155 ubiquitination quantitation

Mouse myocardial tissue samples were lysed using 1% SDS buffer (Tris pH 7.5, 0.5 mM EDTA, and 1 mM DTT) and a 10-min boiling followed by Tris-HCL (pH 8.0) saturation. Cells transfected with HA-tagged (HA)-ubiquitin, full length human Myc-PARP1 and mutant Myc-PARP1 plasmids; or full-length human Flag-BAF155 were also lysed as described above. Cell lysates underwent successive incubations with anti-PARP1 or anti-BAF155 antibodies (1 μg/mg of cell lysate; 4°C) and protein A/G (B23202) or anti-Myc (B26302) immunoprecipitation magnetic beads for 12 h.

10. Coimmunoprecipitation

Mouse myocardial tissue samples and cells were lysed by lysis buffer (50 mM Tris, 137 mM NaCl, 1 mM EDTA, 10 mM NaF, 0.1 mM Na_3VO_4, 1% NP-40, 1 mM DTT, and 10% glycerol, pH 7.8) containing protease inhibitors (Bimake). The resulting lysates were incubated with 30 μl of anti-Myc Affinity Gel (B26302, Biotool; 12 h at 4°C or adequate antibodies (1 μg/mg of cell lysate; 2–3 h) and Protein A/G for immunoprecipitation (B23202; Bio tool) at 30 μl for 12 h at 4°C. The immunoprecipitated complexes were separated by SDS-PAGE followed by electro-transfer onto a PVDF membrane. The membrane was blocked (5% bovine serum albumin) for 1h at ambient, followed by successive incubations with primary (4°C, overnight) and secondary (ambient, 1h) antibodies. Ubiquitinated BAF155 and PARP1, were immunoprecipitated with anti-Myc, anti-PARP1 or anti-BAF155 antibodies, respectively, and detection was carried out with anti-HA or anti-UB antibodies. ImageJ v1.46 (National Institutes of Health, USA) was utilized for signal quantification that was then normalized to GAPDH or tubulin expression.

11. Micro-CT

Cardiac cavity was assessed by the section of the mean heart tissue brightness following contrast agent injection into vein. Micro-computed tomography (micro-CT-Imaging skycan 1276, Bruker, Germany) was performed at 55 kV (200 μA), acquiring 280 sections (13.275mm×13.275mm) in 2 min 51 s with steply ro-tating (degree=0.8). The CT images of the heart were opened with data viewer software, and the left ventricle was divided equally at both ends near the apical and the atrial part, and eight cross-sections with equal interval distance between them were taken as the results of our continuous cardiac scans to assess the area of the

heart chambers.

12. Blood pressure measurement

Blood pressure (BP) was measured in conscious mice by the tail-cuff BP measurement system (BP-2010 Series Blood Pressure Meter, Softron, Japan). The blood pressure machine was prepared and preheated to 37℃ in advance, then the sensor was put on the tail of the mouse, and the mouse was placed in a mouse holder for 5 min to acclimate. Next, the blood pressure in the tail was measured three times per mouse. The average of the three pressure measurements represents the recorded mouse blood pressure.

13. Statistical analysis

Data are shown as mean±standard deviation (SD). Homogeneity of variance was assessed by the F- and Brown–Forsythe tests for two and multiple groups, respectively. The Shapiro–Wilk test was performed for assessing normality. Data with normal and skewed distributions were compared by Student's t test and Welch's *t*-test, respectively for two groups. One-way ANOVA and two-way ANOVA were carried out for multiple group comparisons involving one and two parameters, respectively, followed by *post hoc* Bonferroni test. The *P* values were appropriately adjusted for multiple comparisons. SPSS 22.0 (SPSS, USA) and GraphPad Prism 8.0 Software (GraphPad, Bethesda, MD, USA) were utilized for statistical analysis, with $P<0.05$ indicating statistical significance.

Supplementary Reference

1. Yi, F. et al. The deacetylation-phosphorylation regulation of SIRT2-SMC1A axis as a mechanism of antimitotic catastrophe in early tumorigenesis. *Sci Adv* **7**, eabe5518 (2021).

2. Zhong, J. et al. Angiotensin-converting enzyme 2 suppresses pathological hypertrophy, myocardial fibrosis, and cardiac dysfunction. *Circulation* **122**, 717–728, 18 p following 728 (2010).

3. Mori, J. et al. Agonist-induced hypertrophy and diastolic dysfunction are associated with selective reduction in glucose oxidation: a metabolic contribution to heart failure with normal ejection fraction. *Circ Heart Fail* **5**, 493–503 (2012).

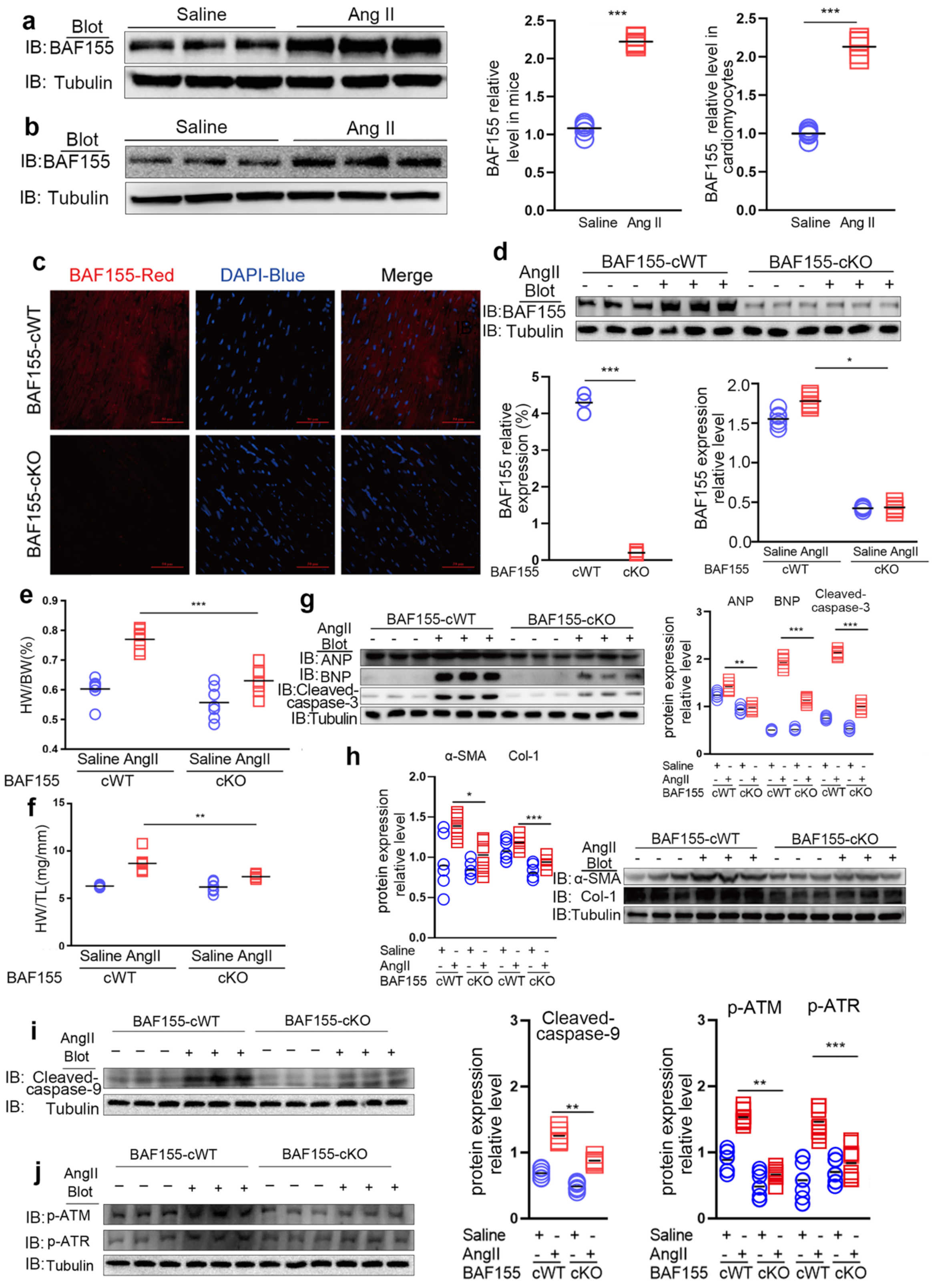

Supplementary Figure S1. BAF155 is up-regulated in failing hearts and cardiomyocytes, myocardium-specific *BAF155* knockout significantly relieves cardiac hypertrophy and fibrosis in mice

a Representative immunoblotting analysis of BAF155 in WT hearts after 2 weeks of Ang II infusion (1.5 mg/kg/day) and quantification of the relative BAF155 protein level. Data are mean±SD (***P<0.001; n=6/group) **b** Representative immunoblotting analysis of BAF155 in H9c2 cells exposed to Ang II (100 nM) for 24h and quantification of the relative BAF155 protein level (***P<0.001; n=6/group). **c** Representative expression levels of BAF155 were examined by immunofluorescence staining. The mouse heart tissues were labeled with anti-BAF155 antibodies (red), and nuclei were labeled with DAPI (blue). Scale bar, 50 μm. Data are mean±SD (***P<0.001; n=6/group). **d** Representative immunoblotting analysis of *BAF155* in the *BAF155*-cWT/cKO mouse heart tissues after 2 weeks of Ang II infusion (1.5 mg/kg/day) and quantification of the relative BAF155 protein level. Data are mean±SD (*P<0.05, n=6/group). **e, f, g** Heart weight/body weight (HW/BW) **(e)** and heart weight/tibia length (HW/TL) **(f)** ratios, and representative immunoblotting **(g)** analysis of ANP, BNP and Cleaved caspase-3, were used to quantitate myocardial hypertrophy. Data are mean±SD (**P<0.01, ***P<0.001; n=6/group). **h** Representative immunoblotting analysis of α-SMA and Col-1 was used to quantify myocardial fibrosis. Data are mean±SD (*P<0.05, ***P<0.001; n=6/group). **i** Representative immunoblotting analysis of Cleaved-caspase-9 was used to quantify and cardiomyocyte death. Data are mean±SD (**P<0.01; n=6/group). **j** Representative immunoblotting analysis of p-ATM and p-ATR was used to quantify DNA damage. Data are mean±SD (**P<0.01, ***P<0.001; n=6/group). Data are expressed as means ± SD. Statistical significance was assessed by 2-way ANOVA with Bonferroni multiple comparisons test (P values adjusted for 6 comparisons, * $P < 0.05$; ** $P < 0.01$; *** $P < 0.001$).

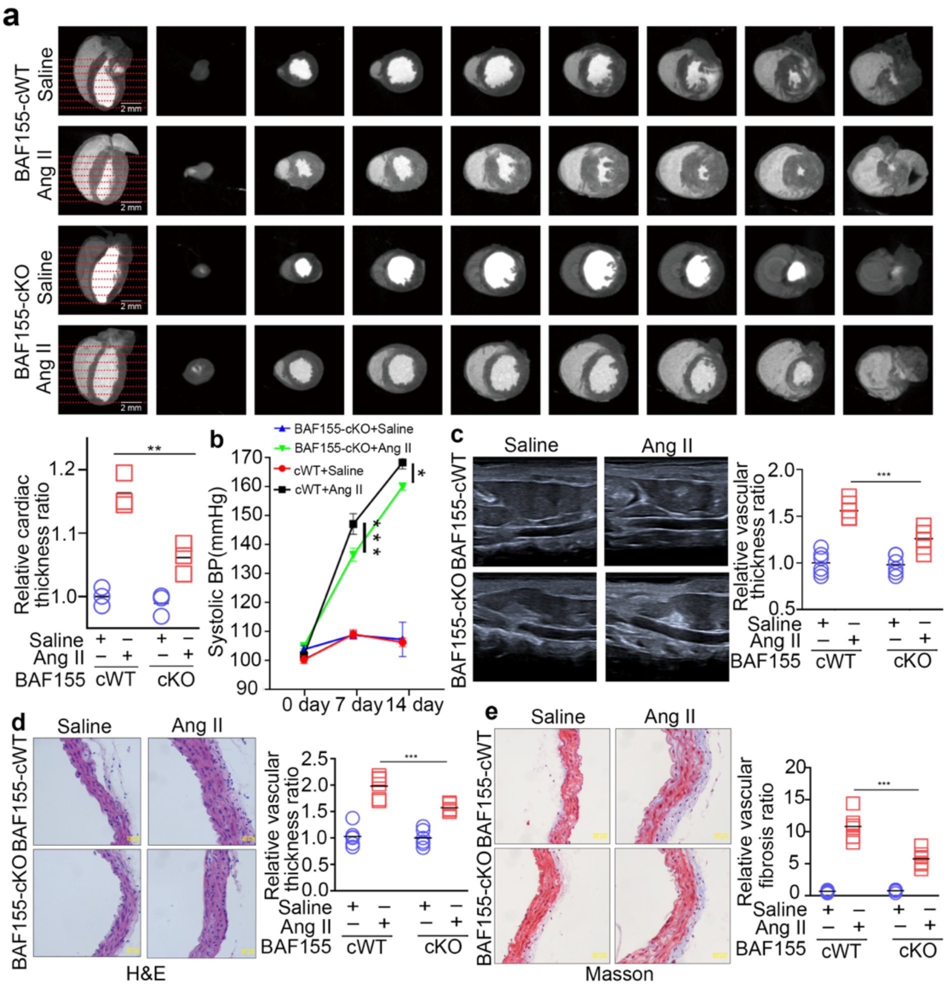

Supplementary Figure S2. myocardium-specific *BAF155* knockout significantly relieves cardiac hypertrophy and vascular thickness and fibrosis in mice

a Plane and selected 3D reconstruction images were used to analyze the cardiac hypertrophy in *BAF155*-cWT mice and *BAF155*-cKO mice after 2 weeks of Ang II infusion (1.5 mg/kg/day). **b** Representative data of blood pressure in *BAF155*-cKO and *BAF155*-cWT mice after 2 weeks of Ang II infusion (1.5 mg/kg/day). Data are mean±SD (**P*<0.05, ****P*<0.001; n=6/group) **c** Representative vascular ultrasound was performed to examine cardiac function changes in *BAF155*-cWT and *BAF155*-cKO mice. mean±SD (****P*<0.001; n=6/group). **d** Representative vascular sections examined by hematoxylin and eosin (H&E) staining. Scale bar, 40μm. Data are mean±SD (****P*<0.001; n=6/group). **e** Representative vascular sections examined by Masson's trichrome staining. Scale bar, 40 μm. Data are mean±SD (****P*<0.001; n=6/group). Data are expressed as means ± SD. Statistical significance was assessed by 2-way ANOVA with Bonferroni multiple comparisons test (*P* values adjusted for 6 comparisons, ** *P* < 0.01; *** *P* < 0.001).

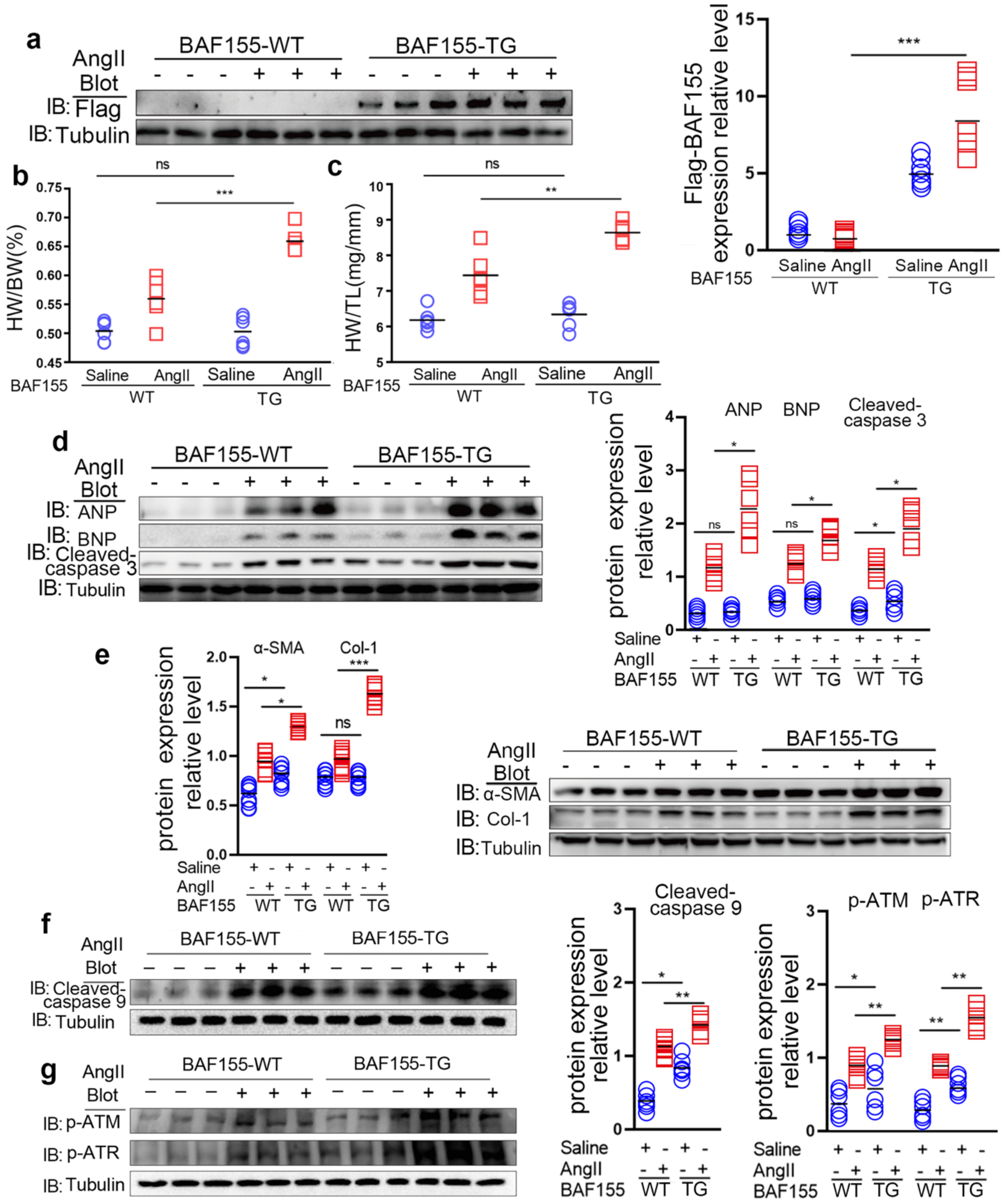

Supplementary Figure S3. BAF155 overexpression significantly aggravates cardiac hypertrophy and fibrosis in mice

a Representative immunoblotting analysis of BAF155 in the *BAF155*-WT/TG mouse heart tissues after 2 weeks of Ang II infusion (1.5 mg/kg/day) and quantification of the relative BAF155 protein level Data are mean±SD (****P*<0.001; n=6/group). **b, c, d** Heart weight/body weight (HW/BW) **(b)** and heart weight/tibia length (HW/TL) **(c)** ratios, and representative

immunoblotting **(d)** analysis of ANP, BNP and Cleaved caspase-3 were used to quantitate myocardial hypertrophy. Data are mean±SD (***P*<0.01, ****P*<0.001; n=6/group). **e** Representative immunoblotting analysis of α-SMA and Col-1 was used to quantify myocardial fibrosis. Data are mean±SD (**P*<0.05, ****P*<0.001; n=6/group). **f** Representative immunoblotting analysis of Cleaved-caspase-9 was used to quantify cardiomyocyte death. Data are mean±SD (**P*<0.05, ***P*<0.01; n=6/group). **g** Representative immunoblotting analysis of p-ATM and p-ATR was used to quantify DNA damage. Data are mean±SD (**P*<0.05, ***P*<0.01; n=6/group). Data are expressed as means ± SD. Statistical significance was assessed by 2-way ANOVA with Bonferroni multiple comparisons test (*P* values adjusted for 6 comparisons, ns *P*>0.05 which means no significance; * $P < 0.05$; ** $P < 0.01$; *** $P < 0.001$).

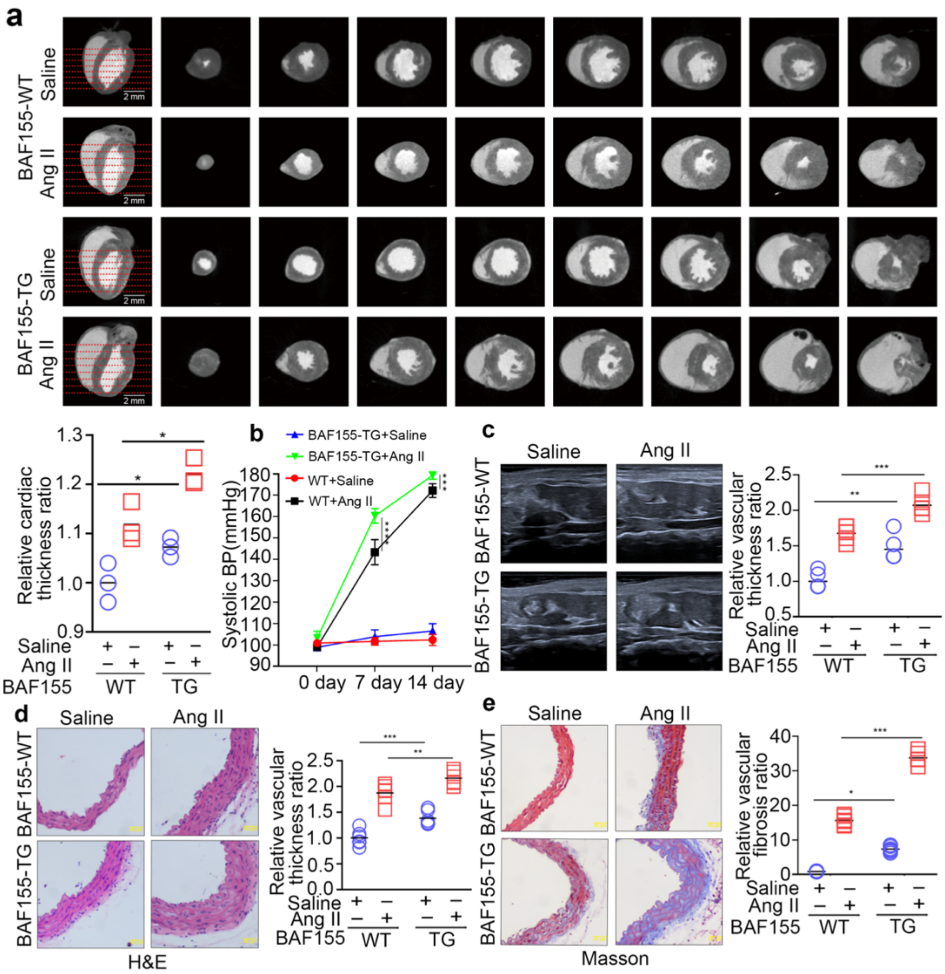

Supplementary Figure S4. BAF155 overexpression significantly aggravates cardiac hypertrophy and vascular thickness and fibrosis in mice

a Plane and selected 3D reconstruction images were used to analyze the cardiac hypertrophy in *BAF155*-WT mice and *BAF155*-TG mice after 2 weeks of Ang II infusion (1.5 mg/kg/day). **b** Representative data of blood pressure in *BAF155*-TG and *BAF155*-WT mice after 2 weeks of Ang II infusion (1.5 mg/kg/day). Data are mean±SD (****P*<0.001; n=6/group). **c** Representative vascular ultrasound was performed to examine the vascular thickness. Data are mean±SD (***P*<0.01, ****P*<0.001; n=6/group). **d** Representative vascular sections of hematoxylin and eosin (H&E) staining. Scale bar, 40 μm. Data are mean±SD (***P*<0.01,****P*<0.001; n=6/group). **e** Masson's trichrome staining to examine the vascular thickness and fibrosis. Scale bar, 40 μm. Data are mean±SD (**P*<0.05, ****P*<0.001; n=6/group). Data are expressed as means ± SD. Statistical significance was assessed by 2-way ANOVA with Bonferroni multiple comparisons test (*P* values adjusted for 6 comparisons, * *P* < 0.05; ** *P* < 0.01; *** *P* < 0.001).

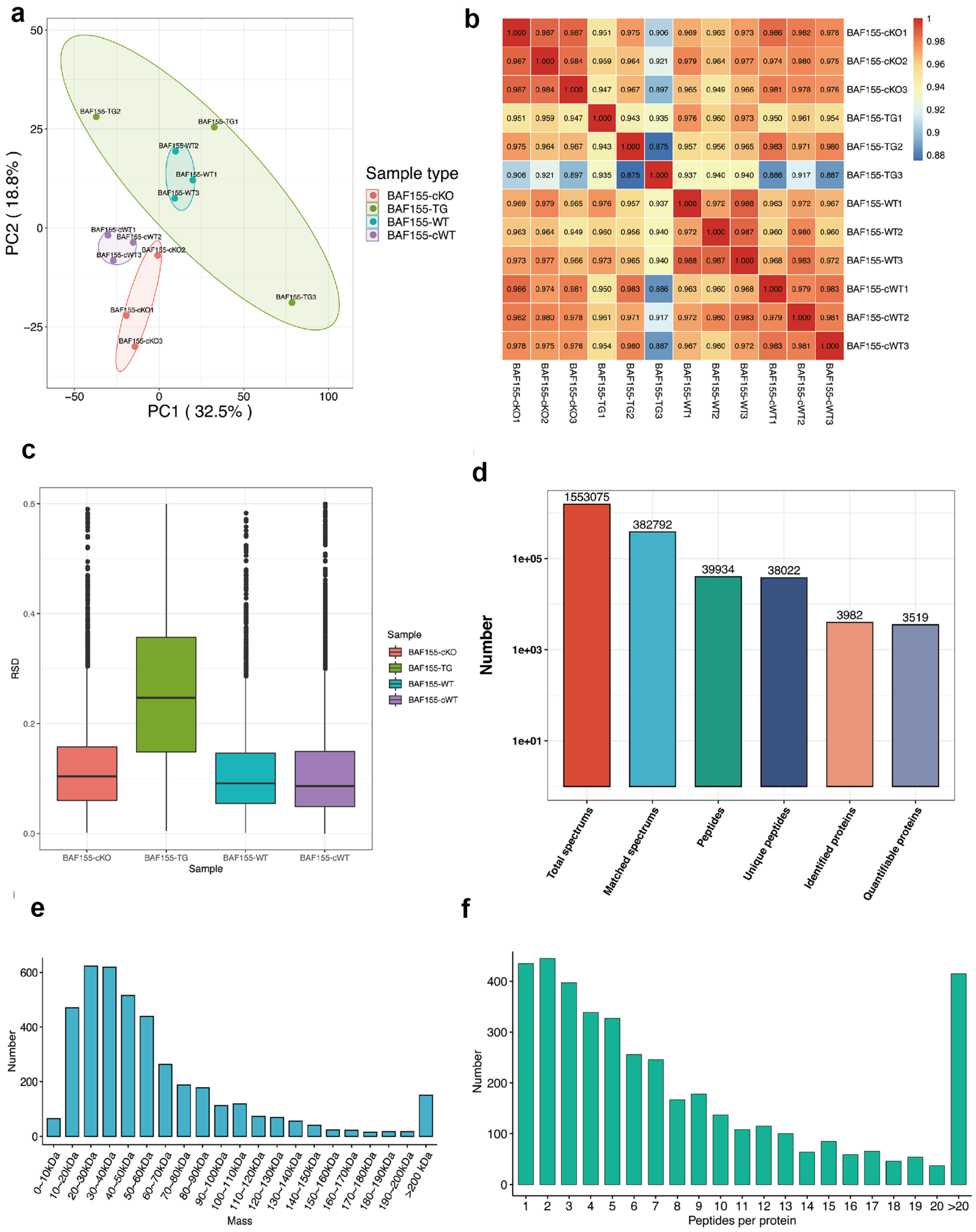

Supplementary Figure S5. Quality control results of the BAF155 proteome

a Principal component analysis (PCA) showing the first two principal components of protein intensities, with specimens linked by centroids based on sample type. **b** Pearson's correlation analysis; each value represents the correlation coefficient between the two samples. **c** A boxplot based on relative standard deviation (RSD), with each point representing an RSD value. **d** Histogram of mass spectrometry results. **e** Protein molecular weight statistics; each bar height represents the protein number. **f** Peptide length statistics after enzymatic hydrolysis, with bar height representing the number of peptides.

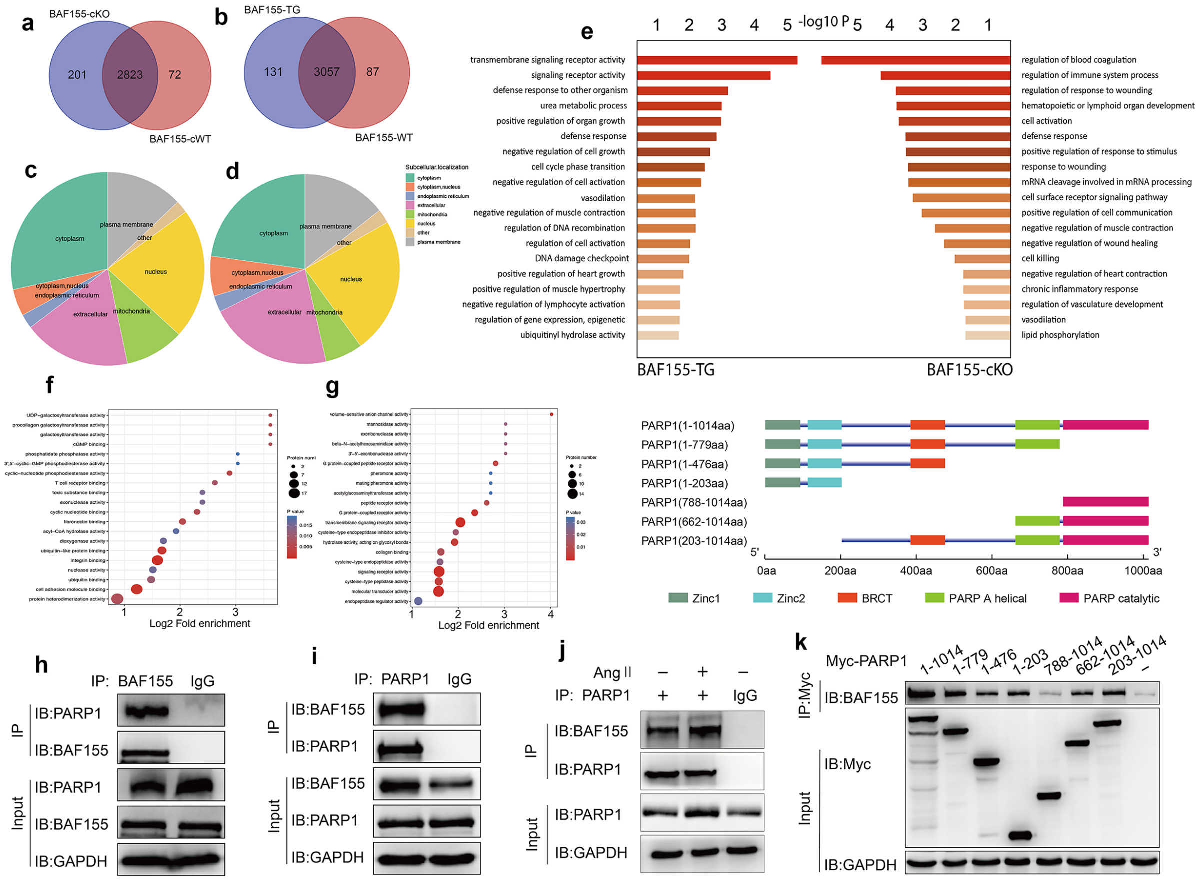

Supplementary Figure S6. Proteomic analysis of differential proteins in *BAF155* knockout and overexpressing mice

a Venn diagram showing identified proteins in mouse heart tissues overlapping between the myocardium-specific *BAF155* knockout (*BAF155*-cKO) and *BAF155*-cWT groups. **b** Venn diagram for the *BAF155* transgenic (*BAF155*-TG) and *BAF155*-WT groups. **c, d** The differentially expressed proteins in the hearts of *BAF155*-cKO and *BAF155*-cWT **(c)** and *BAF155*-TG and *BAF155*-WT **(d)** mice were predicted and classified for subcellular structural mapping (n=3 per group). **e** Biological process enrichment analysis was performed for differentially expressed proteins in *BAF155*-TG and *BAF155*-cKO mice and the corresponding *BAF155*-WT/cWT mice, in heart samples, respectively. The bar length reflects Fisher's exact test *P* value (n=3 per group). **f, g** The differentially expressed proteins in the hearts of *BAF155*-cKO and *BAF155*-cWT **(f)** and *BAF155*-TG and *BAF155*-WT **(g)** mice were enriched by molecular functions and represented by bubble graphs. The bubble color and size represent Fisher's exact test *P* value and the amounts of proteins with corresponding functions, respectively (n=3 per group). **h** Endogenous protein interactions were examined in

cardiomyocyte lysates immunoprecipitated (IP) with anti-BAF155 antibodies or anti-rabbit IgG, and analyzed by Western blot with antibodies to detect BAF155 and PARP1. **i** Endogenous protein interactions were examined in cardiomyocyte lysates immunoprecipitated with anti-PARP1 antibodies or anti-rabbit IgG and analyzed by Western blot with antibodies to detect BAF155 and PARP1. **j** Endogenous protein interactions were examined in cardiomyocyte lysates immunoprecipitated with anti-PARP1 antibodies or anti-rabbit IgG, and analyzed by Western blot with antibodies to detect BAF155 and PARP1 after Saline or Ang II administration for two weeks. **k** Full-length Myc-PARP1 and six truncated Myc-PARP1 plasmids were transfected, respectively, and the total lysate was assessed by immunoprecipitation with anti-Myc antibodies, with subsequent immunoblot detection with anti-BAF155 antibodies.

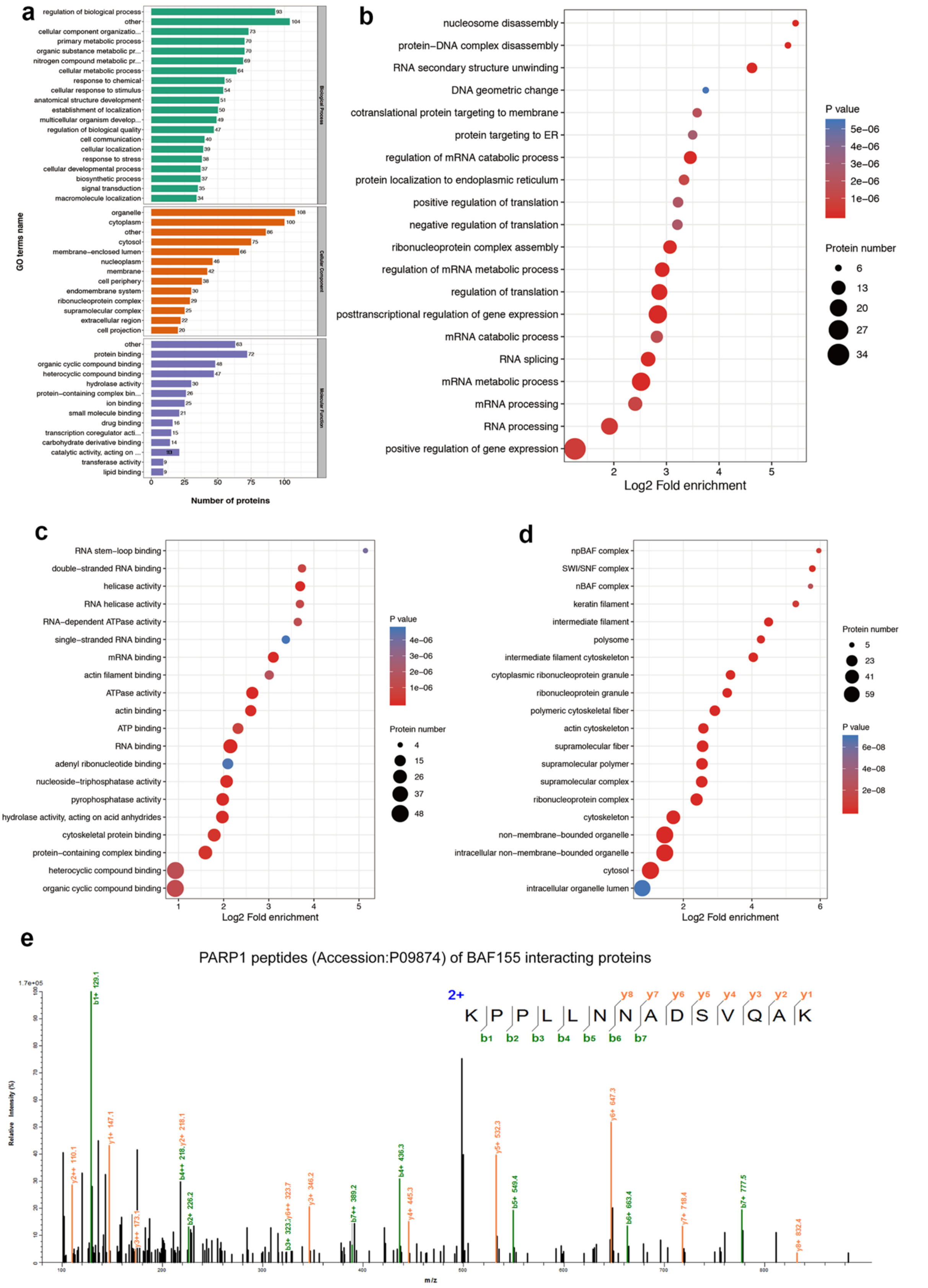

Supplementary Figure S7. Mass spectrometric analysis of differential BAF155-binding proteins

a Gene Ontology classification of BAF155-binding proteins; bar length represents protein number. **b, c, d** Bubble graphs show the Biological Process **(b)**, Molecular Function **(c)**, and Cellular Component enrichment **(d)** for BAF155-binding proteins. The bubble color represents Fisher's exact test *P* value; the bubble size represents the amounts of proteins with corresponding functions. **e** The spectrograms showed mass spectroscopy identified PARP1 peptides (Accession: P09874; Description: Poly [ADP-ribose] polymerase 1 OS=Homo sapiens OX=9606 GN=PARP1 PE=1 SV=4) of BAF155 interacting proteins.

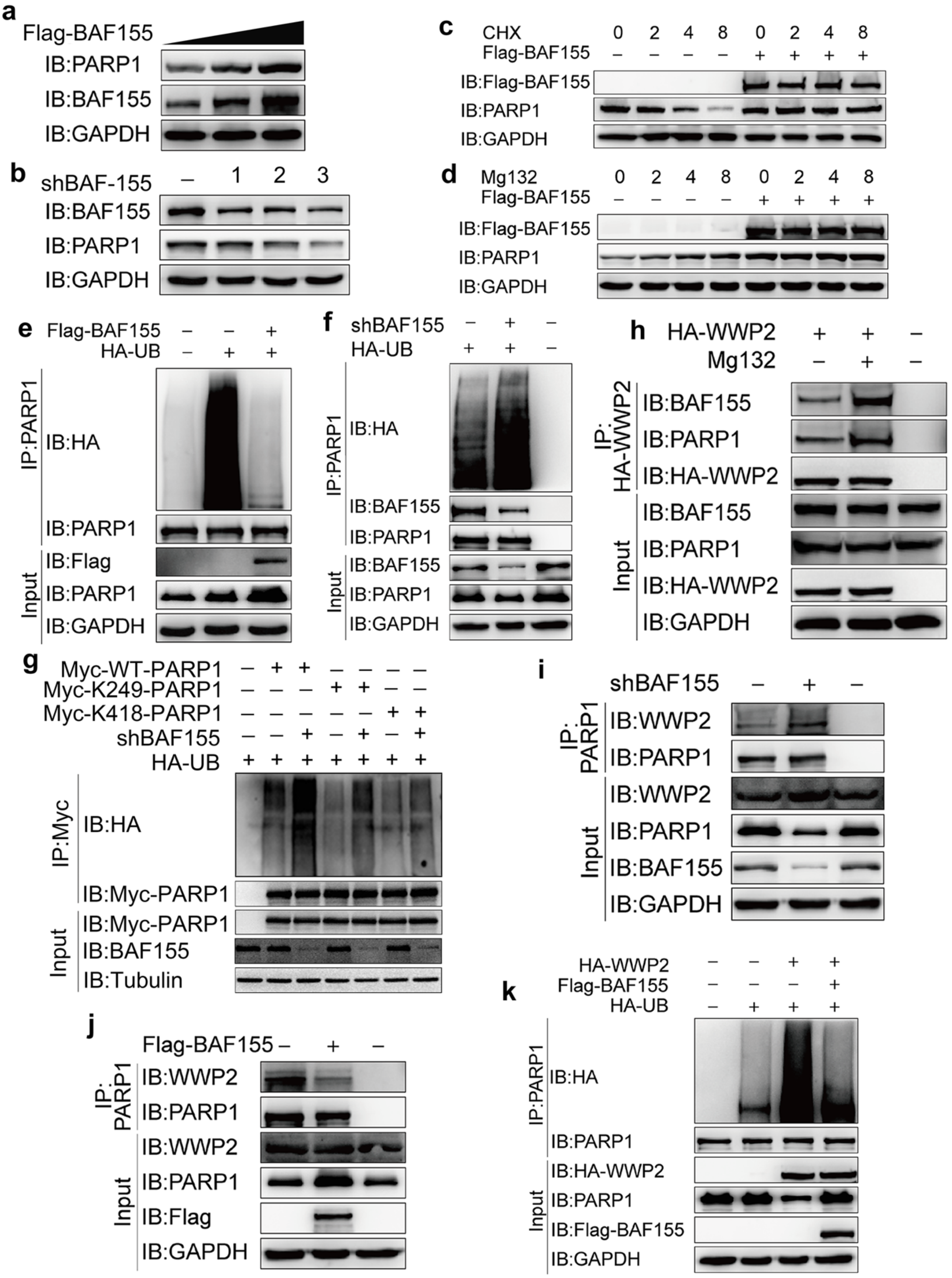

Supplementary Figure S8. BAF155 stabilizes PARP1 and reduces PARP1-K249/K418 ubiquitination by restraining WWP2

a Representative immunoblotting analysis of PARP1 expression level after diverse amounts of the Flag-BAF155 plasmid introduced in cells. **b** Representative immunoblotting analysis of PARP1 expression level was assessed in negative control (NC), small hairpin RNA targeting BAF155 (sh *BAF155*)-56437, sh *BAF155*-56438, or sh *BAF155*-56439 cells. **c** Representative immunoblotting analysis of PARP1 protein expression level in Flag-control and Flag-BAF155 cells underwent subsequent treatment with cycloheximide (CHX) at different time points. **d** Representative immunoblotting analysis of PARP1 protein expression level in Flag-control and Flag-BAF155 cells underwent subsequent treatment with MG132 at different time points. **e** Cells were co-transfected with Flag-BAF155 and HA-UB, and ubiquitinated PARP1 was immunoprecipitated with anti-PARP1 antibodies, followed by immunoblot detection with anti-HA antibodies. **f** NC and shBAF155 H9c2 cells underwent co-transfection with HA-UB, and ubiquitinated PARP1 was immunoprecipitated with anti-PARP1 antibodies, followed by immunoblot detection with anti-HA antibodies. **g** Cells underwent co-transfection with HA-UB, and either Myc-PARP1-WT, Myc-PARP1-K249R, or Myc-PARP1-K418R in the context of normal or knockdown BAF155 levels, and ubiquitinated PARP1 was immunoprecipitated with anti-Myc antibodies, with subsequent detection with anti-HA antibodies. **h** Cells co-transfected with HA-WWP2 were subsequently treated with or without Mg132, followed by immunoprecipitation with anti-HA antibodies and detection with anti-BAF155 and anti-PARP1 antibodies, respectively. **i** NC and shBAF155 H9c2 cells underwent immunoprecipitation with anti-PARP1 antibodies and detection with anti-WWP2 antibodies. **j** Cells underwent co-transfection with or without Flag-BAF155, followed by immunoprecipitation with anti-PARP1 antibodies and detection with anti-WWP2 antibodies, respectively. **k** Cells underwent co-transfection with or without HA-WWP2, Flag-BAF155, and HA-UB, and ubiquitinated PARP1 underwent immunoprecipitation with anti-PARP1 antibodies, followed by detection with anti-HA antibodies.

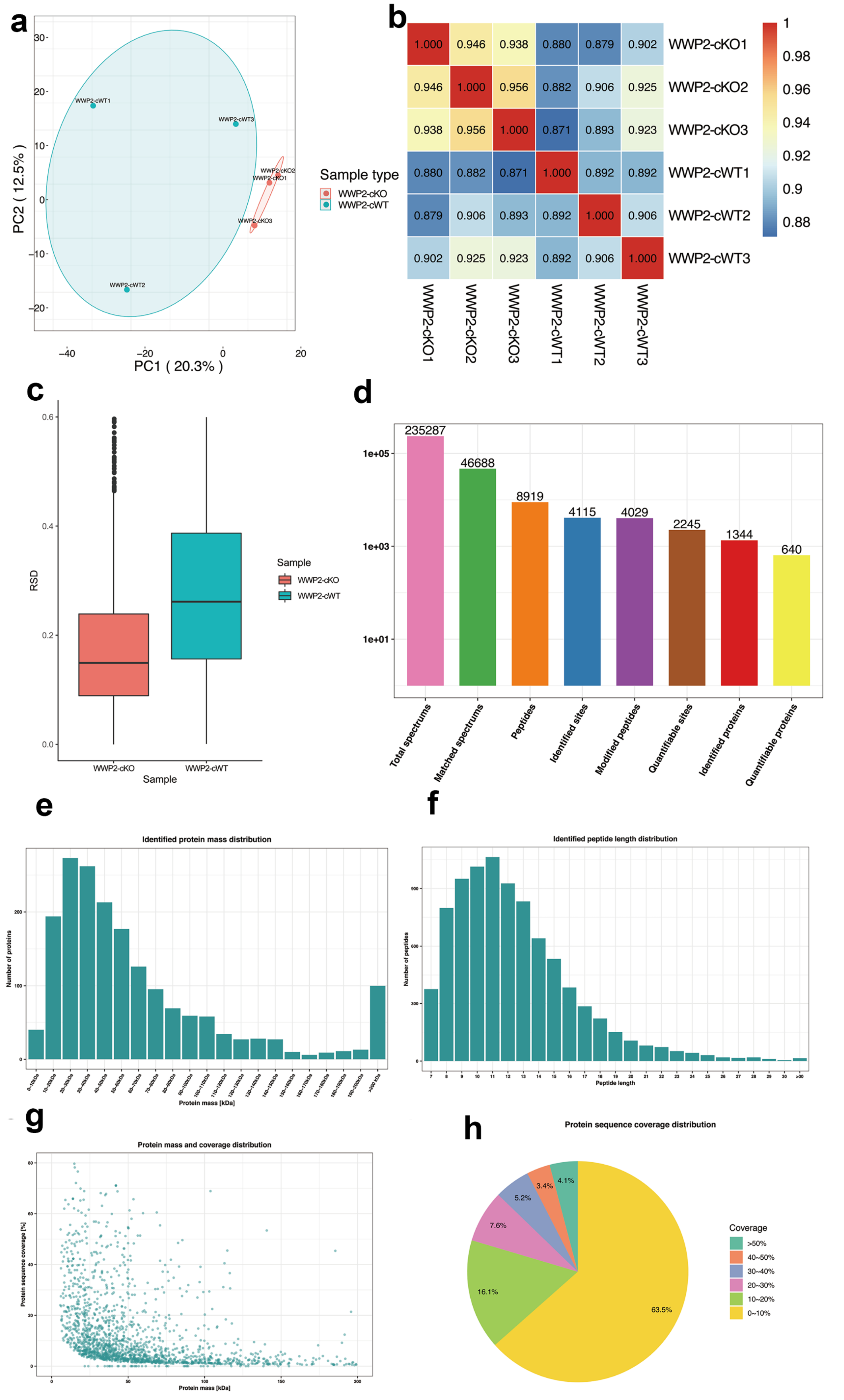

Supplementary Figure S9. Quality control results of the WWP2 ubiquitin proteome

a Principal component analysis (PCA) showing the first two principal components of protein intensities, with specimens linked by centroids based on sample type. **b** Pearson's correlation analysis; each value represents the correlation coefficient between the two samples. **c** A boxplot based on relative standard deviation (RSD), with each point representing an RSD value. **d** Histogram of mass spectrometry results. **e** Protein molecular weight statistics; each bar height represents the protein number. **f** Peptide length statistics after enzymatic hydrolysis, with bar height representing the number of peptides. **g** Relationship between protein coverage and molecular weight; dots indicate the corresponding proteins. **h** Pie charts showing protein coverage.

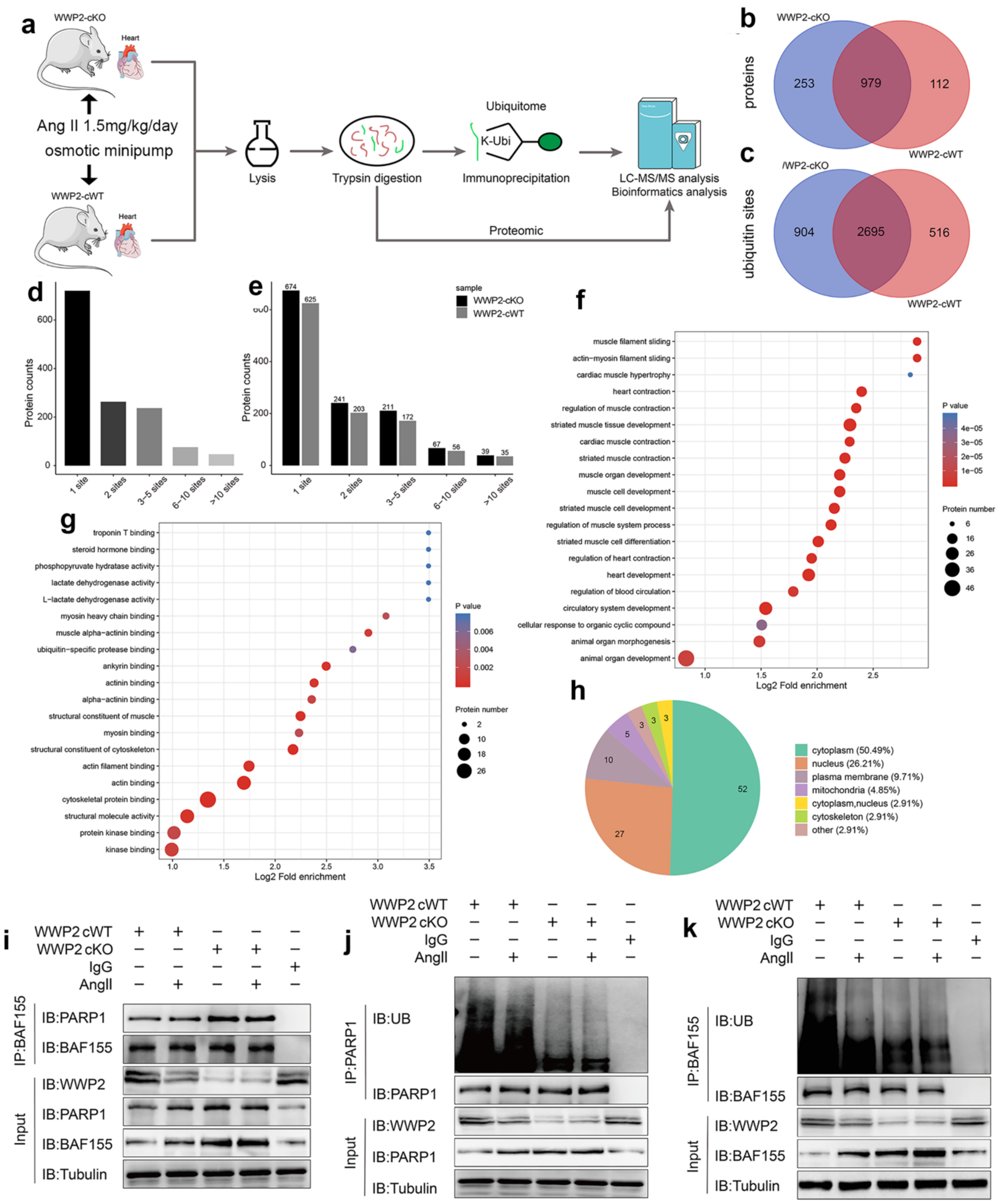

Supplementary Figure S10. Ubiquitination proteomic analysis of differential proteins in myocardium-specific WWP2 knockout mice

a Workflow chart showing the quantitative analysis of the proteome profiles and ubiquitination modifications in myocardium-specific *WWP2* knockout (*WWP2*-cKO) and the corresponding wild-type (*WWP2*-cWT) mice, in heart samples. **b, c** Venn chart showing identified proteins **(b)** and ubiquitin sites **(c)** overlapping in mouse heart tissues between the *WWP2*-cKO and *WWP2*-

cWT groups. **d** Column chart showing the distribution of ubiquitin sites for each protein with differential expression. **e** Column chart showing the distribution of ubiquitin sites identified in heart tissues from *WWP2*-cKO and *WWP2*-cWT mice, respectively. **f** The differentially ubiquitinated proteins in the hearts of *WWP2*-cKO and *WWP2*-cWT mice were enriched by biological process and represented by a bubble graph. The bubble color and size represent Fisher's exact test *P* value and the amounts of proteins with corresponding functions, respectively (n=3 per group). **g** The differentially ubiquitinated proteins in the hearts of *WWP2*-cKO and *WWP2*-cWT mice were enriched by molecular function and represented by a bubble graph. The bubble color and size represent Fisher's exact test *P* value and the amounts of proteins with corresponding functions, respectively (n=3 per group). **h** The differentially ubiquitinated proteins in the hearts of *WWP2*-cKO and *WWP2*-cWT mice were predicted and classified for subcellular structural mapping, (n=3 per group). **i** *WWP2*-cWT and *WWP2*-cKO heart tissues were obtained from mice with or without Ang II treatment, and underwent immunoprecipitation with anti-BAF155 antibodies, followed by detection with anti-PARP1 antibodies. **j** *WWP2*-cWT and *WWP2*-cKO heart tissues were obtained from mice with or without Ang II treatment, and ubiquitinated PARP1 underwent immunoprecipitation with anti-PARP1 antibodies, followed by detection with anti-UB antibodies. **k** *WWP2*-cWT and *WWP2*-cKO heart tissues were obtained from mice with or without Ang II treatment, and ubiquitinated BAF155 underwent immunoprecipitation with anti-BAF155 antibodies, followed by detection with anti-UB antibodies.

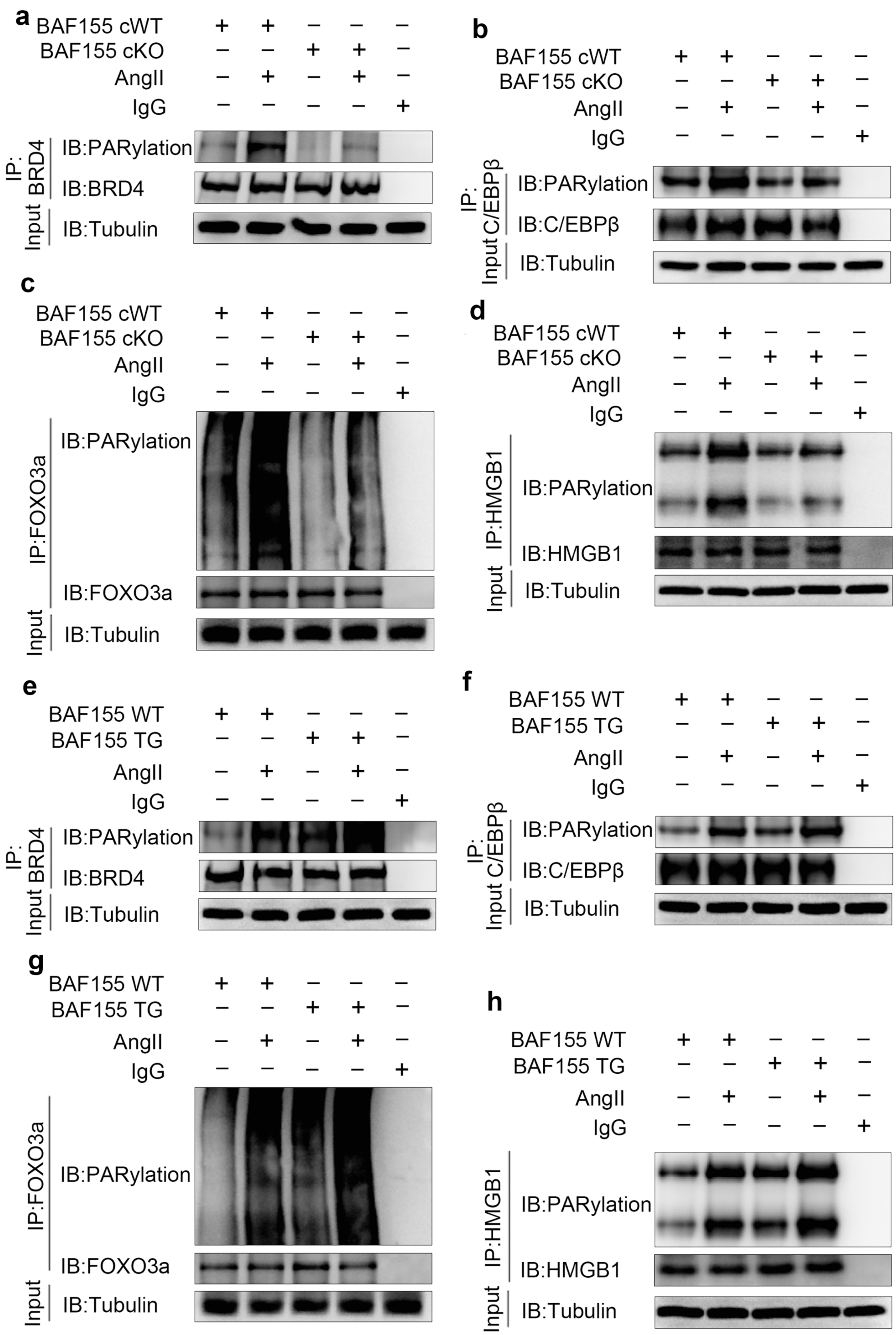

Supplementary Figure S11. BAF155 increased the PARylation modification of BRD4, CEBPβ, FOXO3a and HMGB1

a,b,c,d Representative immunoblotting analysis of the level of PARylation in BRD4 **(a)**, CEBPβ **(b)**, FOXO3a **(c)** and HMGB1 **(d)** in *BAF155*-cKO and *BAF155*-cWT mice. **e,f,g,h** Representative immunoblotting analysis of the level of PARylation in BRD4 **(e)**, CEBPβ**(f)**, FOXO3a **(g)** and HMGB1 **(h)** in *BAF155*-TG and *BAF155*-WT mice.

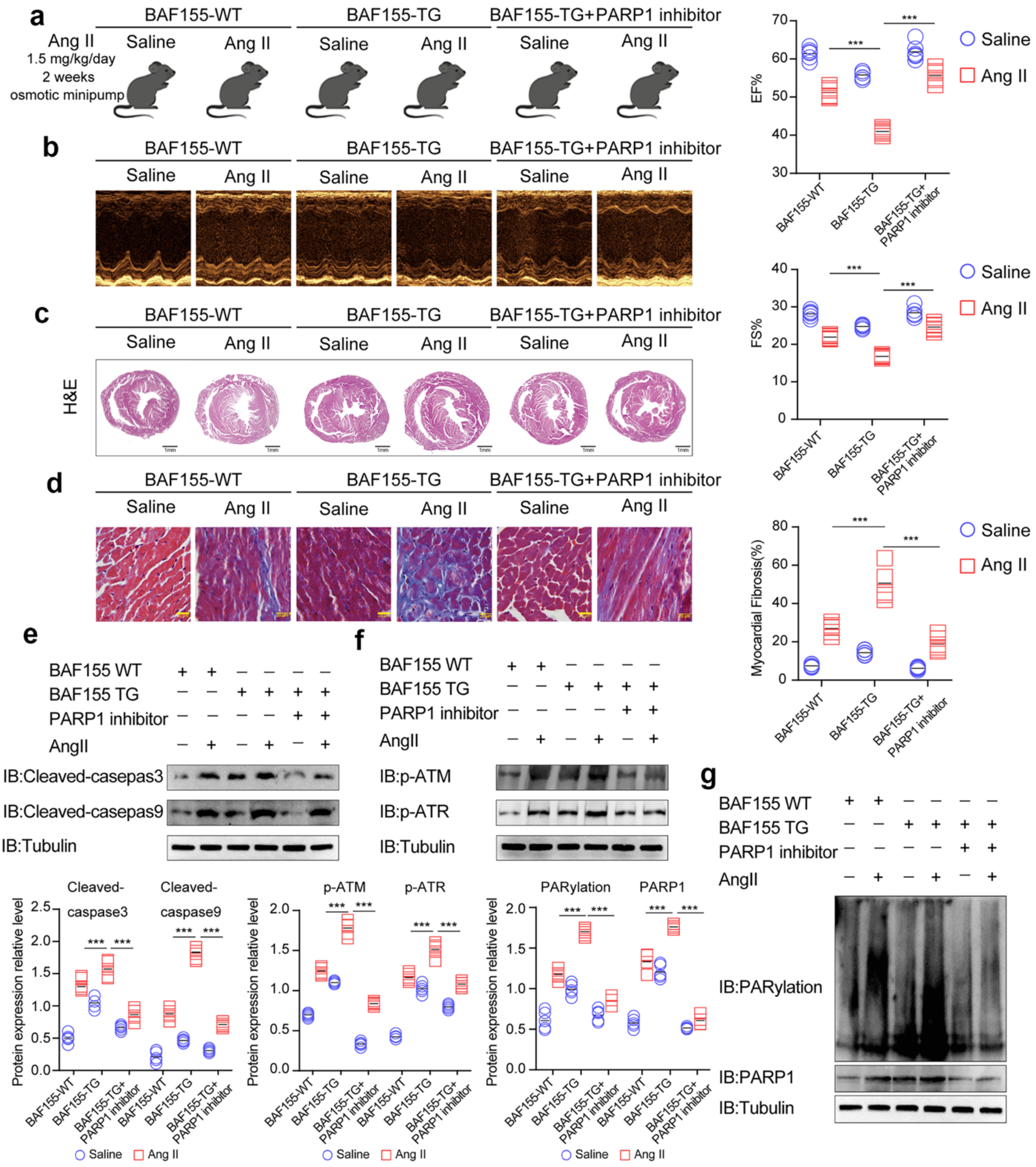

Supplementary Figure S12. PARP1 inhibitor restrain the detrimental effects of BAF155 overexpression on cardiac hypertrophy and fibrosis in mice

a *BAF155*-WT and *BAF155* transgenic (*BAF155*-TG) mice were administered control vehicle (Saline), Ang II (1.5 mg/kg/day) through a subcutaneously implanted osmotic minipump at 0.5 μl/h or PARP1 inhibitors (ABT-888) injected intraperitoneally (25mg/kg/day) for 2 weeks, followed by euthanasia. **b** Representative echocardiography of the left ventricular chamber was

performed to examine cardiac function changes in *BAF155*-WT and *BAF155*-TG mice after administered Ang II and PARP1 inhibitor separately. Ejection fraction (EF%) and fractional shortening (FS%) are expressed as mean±SD (****P*<0.001; n=6/group). **c** Representative heart sections of hematoxylin and eosin (H&E) staining after administered Ang II and PARP1 inhibitor separately to examine the cardiac hypertrophy. Scale bar, 1mm. **d** Masson's trichrome staining in *BAF155*-WT and *BAF155*-TG mice after administered Ang II and PARP1 inhibitor separately to examine the cardiac fibrosis. Scale bar, 20 µm. Data are mean±SD (****P*<0.001; n=6/group). **e** Representative immunoblotting analysis of Cleaved caspase-3, Cleaved-caspase-9 was used to quantify cardiomyocyte death in *BAF155*-WT and *BAF155*-TG mice after administered Ang II and PARP1 inhibitor separately. Data are mean±SD (****P*<0.001; n=6/group). **f** Representative immunoblotting analysis of p-ATM and p-ATR was used to quantify DNA damage in *BAF155*-WT and *BAF155*-TG mice after administered Ang II and PARP1 inhibitor separately. Data are mean±SD (****P*<0.001; n=6/group). **g** Representative immunoblotting analysis of PARP1 and PARylation in *BAF155*-WT and *BAF155*-TG mice after administered Ang II and PARP1 inhibitor separately. Data are mean±SD (****P*<0.001; n=6/group). Data are expressed as means ± SD. Statistical significance was assessed by 2-way ANOVA with Bonferroni multiple comparisons test (*P* values adjusted for 10 comparisons, *** *P* < 0.001).

Supplementary Table S1. Proteins interact with BAF155

Accession	Description	MW [kDa]
P35579	Myosin-9 OS=Homo sapiens OX=9606 GN=MYH9 PE=1 SV=4	226.4
P35580	Myosin-10 OS=Homo sapiens OX=9606 GN=MYH10 PE=1 SV=3	228.9
Q58EY4	SWI/SNF related, matrix associated, actin dependent regulator of chromatin, subfamily c, member 1 OS=Homo sapiens OX=9606 GN=SMARCC1 PE=2 SV=1	122.8
B4DW52	cDNA FLJ55253, highly similar to Actin, cytoplasmic 1 OS=Homo sapiens OX=9606 PE=2 SV=1	38.6
V9HVZ7	Epididymis luminal protein 176 OS=Homo sapiens OX=9606 GN=HEL-176 PE=2 SV=1	25
Q7Z406	Myosin-14 OS=Homo sapiens OX=9606 GN=MYH14 PE=1 SV=2	227.7
P51532	Transcription activator BRG1 OS=Homo sapiens OX=9606 GN=SMARCA4 PE=1 SV=2	184.5
Q8TAQ2	SWI/SNF complex subunit SMARCC2 OS=Homo sapiens OX=9606 GN=SMARCC2 PE=1 SV=1	132.8
B4DI39	cDNA FLJ54328, highly similar to Heat shock 70 kDa protein 1 OS=Homo sapiens OX=9606 PE=2 SV=1	67.5
Q13707	ACTA2 protein (Fragment) OS=Homo sapiens OX=9606 GN=ACTA2 PE=3 SV=1	36.8
Q12824	SWI/SNF-related matrix-associated actin-dependent regulator of chromatin subfamily B member 1 OS=Homo sapiens OX=9606 GN=SMARCB1 PE=1 SV=2	44.1
P22626	Heterogeneous nuclear ribonucleoproteins A2/B1 OS=Homo sapiens OX=9606 GN=HNRNPA2B1 PE=1 SV=2	37.4
G5E975	SWI/SNF-related matrix-associated actin-dependent regulator of chromatin subfamily B member 1 OS=Homo sapiens OX=9606 GN=SMARCB1 PE=1 SV=1	45
P09874	*Poly [ADP-ribose] polymerase 1 OS=Homo sapiens OX=9606 GN=PARP1 PE=1 SV=4	113
Q92925	SWI/SNF-related matrix-associated actin-dependent regulator of chromatin subfamily D member 2 OS=Homo sapiens OX=9606 GN=SMARCD2 PE=1 SV=3	58.9
Q96GM5	SWI/SNF-related matrix-associated actin-dependent regulator of chromatin subfamily D member 1 OS=Homo sapiens OX=9606 GN=SMARCD1 PE=1 SV=2	58.2
Q6FI97	BAF53A protein OS=Homo sapiens OX=9606 GN=BAF53A PE=2 SV=1	47.4
P27348	14-3-3 protein theta OS=Homo sapiens OX=9606 GN=YWHAQ PE=1 SV=1	27.7
B4DN41	DEAD box protein 5 OS=Homo sapiens OX=9606 PE=2 SV=1	67.7

Supplementary Table S2. Plasmids and shRNA used in this study

PLASMID	SPECIES	TAG	VECTOR	IDENTIFIER	SOURCE
WT-BAF155	Human	Flag	GV141	GOSE84500	GeneChem (China)
WT-PARP1 (1-1014)	Human	Myc	GV219	GOSE0175899	GeneChem (China)
PARP1(1-779)	Human	Myc	GV219	GOSE0165581	GeneChem (China)
PARP1(203-1014)	Human	Myc	GV219	GOSE0174296	GeneChem (China)
PARP1(1-476)	Human	Myc	SV40		Sangon Biotech (China)
PARP1(1-203)	Human	Myc	SV40		Sangon Biotech (China)
PARP1(476-1014)	Human	Myc	SV40		Sangon Biotech (China)
PARP1(779-1014)	Human	Myc	SV40		Sangon Biotech (China)
K249R-PARP1	Human	Myc	GV219	GOSE0175903	GeneChem (China)
K418R-PARP1	Human	Myc	GV219	GOSE0175907	GeneChem (China)
Ub	Human	HA	SV40		Sangon Biotech (China)
WWP2	Human	HA	SV40		Sangon Biotech (China)
**shRNA**	**SPECIES**	**TARGET SEQUENCE**			**SOURCE**
sh *BAF155* 56437	Rat	AAGGATATCATCAAACGGCAT			GeneChem (China)
sh *BAF155* 56438	Rat	TGGGAAGCGTCGAAATCAGAA			GeneChem (China)
sh *BAF155* 56439	Rat	GAGCTCACTGATACATGTAAA			GeneChem (China)

Supplementary Table S3. Antibodies and reagents used in this study

ANTIBODY	IDENTIFIER	SOURCE
Anti-BAF155	Cat#NBP2-20415	Novus Biologicals (USA)
Anti-WWP2	Cat#ab103527	Abcam (USA)
Anti-UB	Cat#58395s	Cell Signaling Technology (USA)
Anti-Myc	Cat#2276	Cell Signaling Technology (USA)
Anti-HA	Cat#3724	Cell Signaling Technology (USA)
Anti-PARP1	Cat#9532	Cell Signaling Technology (USA)
Anti-Cleaved-caspase3	Cat#9664	Cell Signaling Technology (USA)
Anti-BRD4	Cat#13440	Cell Signaling Technology (USA)
Anti-foxO3a	Cat#12829	Cell Signaling Technology (USA)
Anti-C/EBPβ	Cat#43095	Cell Signaling Technology (USA)
Anti-HMGB1	Cat#6893	Cell Signaling Technology (USA)
Anti-Phospho-ATR	Cat#2853	Cell Signaling Technology (USA)
Anti-Phospho-ATM	Cat#5883	Cell Signaling Technology (USA)
Anti-Cleaved-caspase9	Cat#20750	Cell Signaling Technology (USA)
Anti-PARylation	Cat#4335-MC-100	Trevigen (USA)
Anti-Flag	Cat#GNI4110-FG	GNI (Japan)
Anti-Myc	Cat#GNI4110-MC	GNI (Japan)
Anti-HA	Cat#GNI4110-HA-P	GNI (Japan)
Anti-GAPDH	Cat#10494-1-AP	Proteintech (China)
Anti-Tubulin	Cat#11224-1-AP	Proteintech (China)
Anti-ANP	Cat#DF6497	Affbiotech(USA)
Anti-BNP	Cat#DF6902	Affbiotech(USA)
Anti-α-SMA	Cat#14395-1-AP	Proteintech (China)
Anti-Col-1	Cat#14695-1-AP	Proteintech (China)
**REAGENT**		
Masson's Trichrome Stain Kit	Cat#G1340	Solarbio (China)
ROS Assay Kit	Cat# C1300-2	Applygen Technologies Inc(China)
ROS Assay Kit	Cat#S0033	Beyotime Biotechnology (China)
WGA Assay Kit	Cat#GTX01502	Genetex (USA)
Fetal Bovine Serum	Cat#FB15015	Clark (USA)
High-glucose DMEM	Cat#01-055-1A	Biological Industries (USA)
Lipofectamine 3000	Cat#L3000150	Invitrogen (USA)
MG132	Cat#A2585	Apexbio (USA)
CHX	Cat#A8244	Apexbio (USA)
Ang II	Cat#A9525	Sigma (USA)
Veliparib-PARP1 inhibitor	Cat#S1004	Selleck (USA)
Protease Inhibitor	Cat#B14002	Bimake (USA)
Anti-Myc magnetic beads	Cat#B26302	Bimake (USA)
Protein A/G magnetic beads	Cat#B23202	Bimake (USA)
DAB plus	Cat#DAB-2031	MXB Biotechnologies (China)
UltraSensitiveTM SP(Mouse/Rabbit) IHC Kit	Cat#KIT-9710	MXB Biotechnologies (China)
Tris-EDTA Antigen Retrieval Solution,10×(ph=9.0)	Cat#C1038	Solarbio (China)
